# Supplementary material for: Reconstituted ovaries self-assemble without an ovarian surface epithelium
Source: Stem Cell Reports. 2023 Oct 26;18(11):2190–202. doi: 10.1016/j.stemcr.2023.10.001 (PMC10679655; doi:10.1016/j.stemcr.2023.10.001)
Supplement: Document S1. Figures S1–S3 and Tables S1–S5 [file mmc1.pdf]

Stem Cell Reports, Volume *18*

Supplemental Information

Reconstituted ovaries self-assemble without an ovarian surface epithelium

Enrique Sosa, Sinthia Kabir Mumu, Christian C. Alvarado, Qiu Ya Wu, Isaias Roberson, Alejandro Espinoza, Fei-man Hsu, Kaori Saito, Timothy J. Hunt, Jared E. Faith, Matthew G. Lowe, Jonathan A. DiRusso, and Amander T. Clark

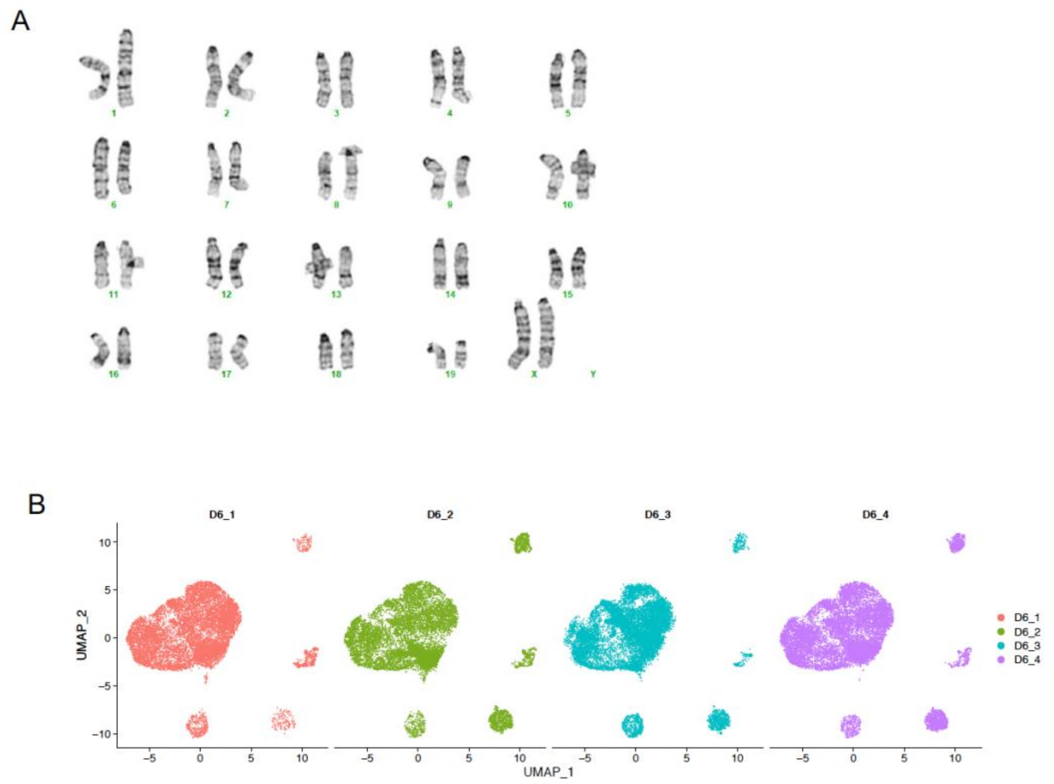

Figure S1: Characterization of PGCLCs and somatic cells. Related to Figure 1.

A) Karyotype with G-banding of the BVSCs iPSC clone 4 FR C3 used for PGCLC and somatic cell differentiation.

B) UMAP of the results from each Day 6 differentiation experiment shown separately indicating equal contribution of cells from each experiment to the five distinct cell populations.

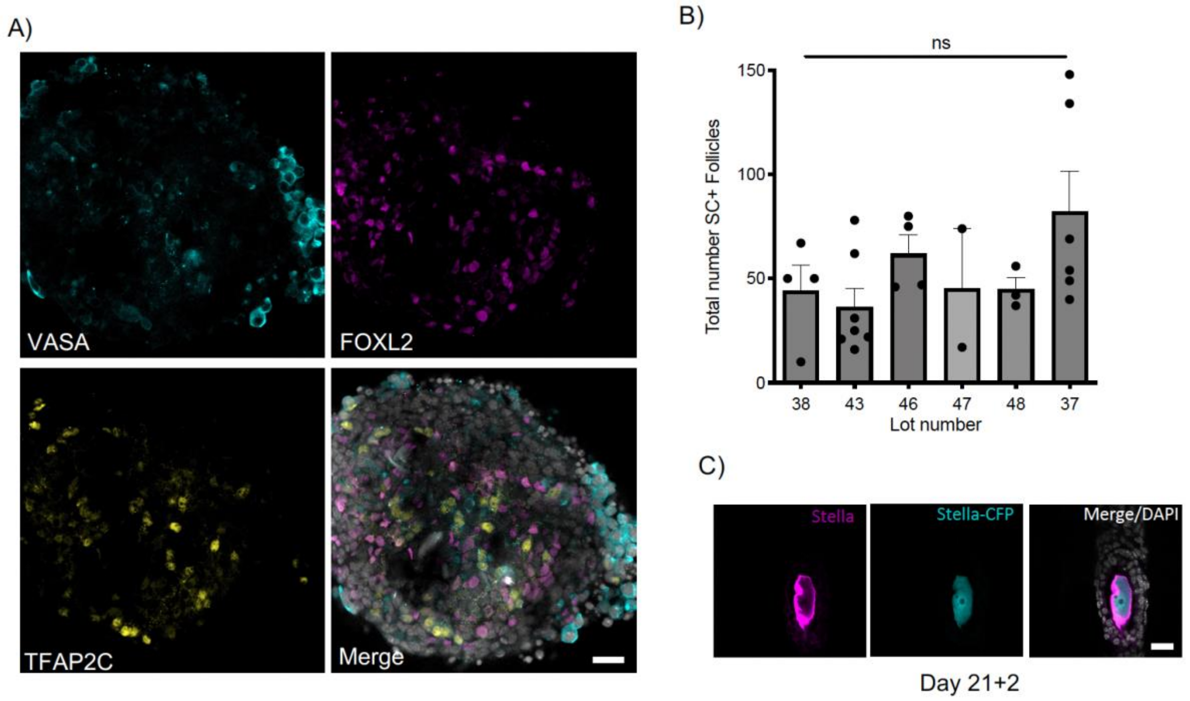

Figure S2: Characterization of rOvaries. Related to Figure 4.

A) IF of aggregates containing FOSCs and PGCLCs at day 2 of aggregation. TFAP2C (yellow) marks the PGCLCs. VASA (blue) marks the endogenous PGCs that escape MACS. FOXL2 (pink) marks pre-granulosa/granulosa cells. Scale bar is 10um.

B) Average number of CFP+ oocytes in rOvaries at the conclusion of IVDi. X-axis refers to different Lots of FOSCs. Each dot refers to the number of SC+ oocytes in a given ovary. Data are shown as mean and standard error of the mean. Statistical analysis in E and F involved an *ordinary one-way ANOVA followed by Tukey's multiple comparison test. Significance was accepted if p<0.05.*

C) IF for STELLA was performed on frozen sections of day 21 rOvaries so that CFP could still be detected. Scale bar is 20um.

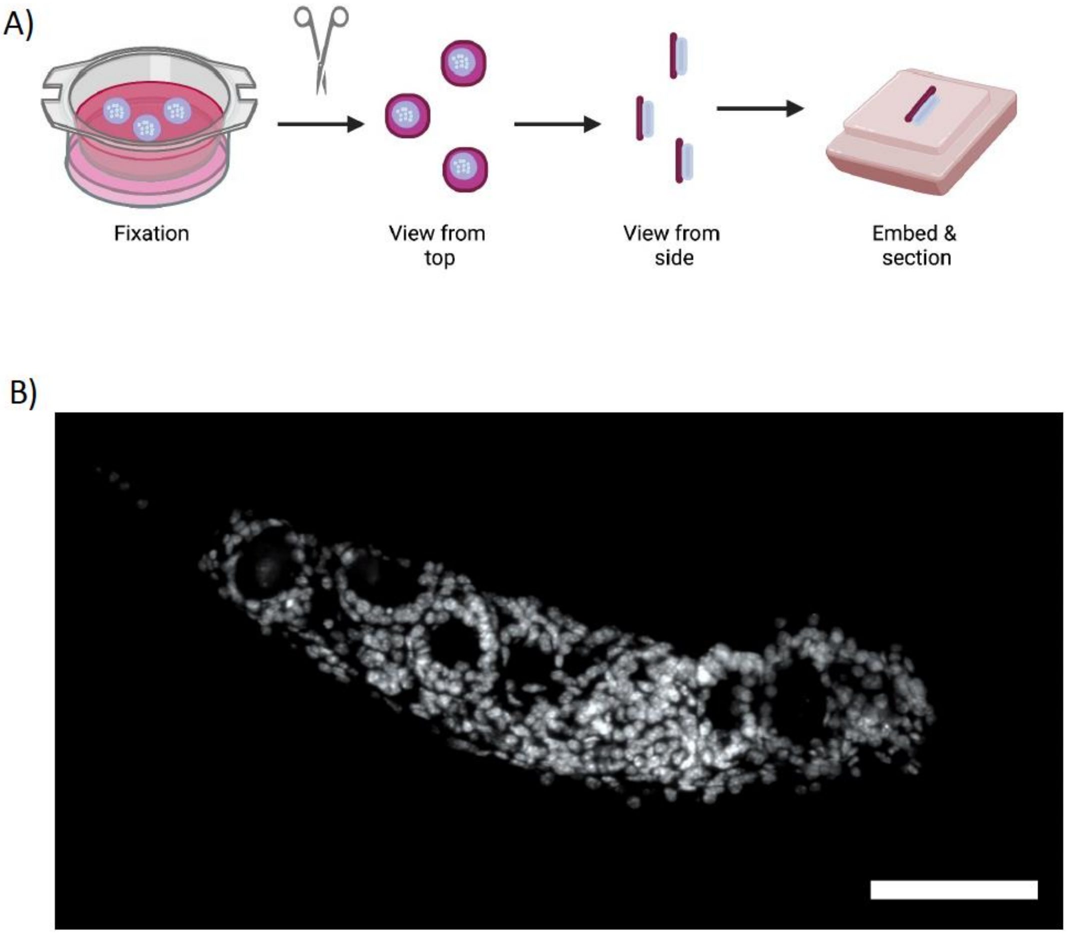

Figure S3: Processing of rOvaries at day 21 of IVDi. Related to Figure 5.

A) Schematic for processing rOvaries at day 21 of IVDi. Created with Biorender.com.
B) DAPI stain of an rOvary showing a view from the side. Scale bar is 100um.

SUPPLEMENTAL TABLES

Table S1: Filtering strategy used to identify valid cells at day 6 of differentiation for further analysis. Related to Figure 1.

Sample	# Reads	Valid Barcodes (%)	# Valid Cells	# Genes per cell	# UMI per cell
D6_1	278,803,599	97.6%	10,419	3,700	10,002
D6_2	1,633,860,575	97.2%	10,290	6,724	41,055
D6_3	179,195,300	97.1	12,289	2,685	6,208
D6_4	109,172,922	97.2	12,194	2,145	4,270

Table S2: Filtering strategy used to identify valid FOSCs for 10X genomics analysis. Related to Figure 2.

FOSC Lot	# Reads	Mappability (%)	# Valid Cells	# Genes per cell	# UMI per cell
Lot 26	389,976,563	76.4	9,668	3,626	11,581
Lot 29	229,173,801	77.4	8,548	3,102	9,322
Lot 30	417,755,755	77.8	10,692	3,686	12,053

Table S3: Percentage and number of Cell types identified in each FOSC Lot. Related to Figure 2.

	Lot 26 (%)	Lot 26 (number)	Lot 29 (%)	Lot 29 (number)	Lot 30 (%)	Lot 30 (number)
Pre-granulosa	46.74%	4519	41.51%	3548	48.77%	5215
Epithelial	17.09%	1652	23.98%	2050	22.40%	2395
Stromal fibroblast	25.19%	2435	25.61%	2189	23.92%	2558
Germ	7.40%	715	3.90%	333	3.41%	365
Endothelial	1.77%	171	1.01%	86	0.80%	86
Macrophage	0.72%	70	0.27%	23	0.41%	44
RBC	0.18%	17	0.40%	34	0.12%	13
Sertoli Cells	0.92%	89	3.33%	285	0.15%	16

Table S4: Total number of CFP-negative oocytes at D21+2 from rOvaries generated with different FOSC Lots. Related to Figure 4.

rOvary	Lot 26	Lot 29	Lot 30	Lot 37	Lot 38	Lot 43	Lot 46	Lot 47	Lot 48
1	9	5	3	11	26	17	11	4	10
2	10	12	9	18	19	6	7	1	9
3	4	13	0	9	26	8	9	1	12
4	10		8	18		5	19	2	
5	8		10	14			14	2	
6	1		13				16	6	
7	5						7		
8	8						10		
9	24						5		
10	12						1		
11	16								

Table S5. List of antibodies used in this study.

Target antigens	Antibodies	Dilutions	Application	Suppliers	Catalog#	RRID
FOXL2	Goat polyclonal anti-FOXL2	1:100	IF	Novus Biologicals	NB100-1277	AB_2106188
KRT19	Rabbit polyclonal anti-KRT19	1:200	IF	Proteintech	10712-1-AP	AB_2133325
LAMININ	Rabbit polyclonal anti-LAMININ	1:200	IF	Abcam	ab11575	AB_298179
NR2F2 (COUP-TF II)	Mouse monoclonal anti-NR2F2	1:200	IF	R&D Systems	PP-H7147-00	AB_2155627
3βHSD	Mouse monoclonal anti-3βHSD	1:200	IF	Cosmo bio	KO607	AB_2722746
TFAP2C (AP-2γ)	Mouse monoclonal anti-AP-2γ	1:200	IF	Santa Cruz Biotech	sc-12762	AB_667770
VASA (DDX4/MVH)	Rabbit poly clonal anti-DDX4/MVH	1:100	IF	Abcam	ab13840	AB_443012

VASA (DDX4)	Goat polyclonal anti-hVASA	1:100	IF	R&D	AF2030	AB_2277369
CFP	Chicken polyclonal anti-GFP	1:200	IF	Abcam	ab13970	AB_300798
Chicken IgY	FITC-conjugated Donkey anti chicken IgY	1:400	IF	Abcam	ab63507	AB_1139472
Mouse IgG	AF488-conjugated donkey-anti-mouse	1:200; 1:400	IF	Jackson ImmunoResearch	715-546-150	AB_2340849
Mouse IgG	AF647-conjugated donkey-anti-mouse	1:200	IF	Jackson ImmunoResearch	715-605-151	AB_2340863
Goat IgG	AF594-conjugated donkey-anti-goat	1:200	IF	Jackson ImmunoResearch	705-586-147	AB_2340434
Rabbit IgG	AF488-conjugated donkey-anti-rabbit	1:200; 1:400	IF	Jackson ImmunoResearch	711-545-152	AB_2313584
SSEA-1(CD15) human, mouse	Anti-SSEA-1(CD15) MicroBeads	1:8	MACS	Miltenyibiotec	130-094-530	AB_2814656
CD31 mouse	Anti-CD31 MicroBeads	1:8	MACS	Miltenyibiotec	130-097-418	AB_2814657
SSEA-1 human, mouse	PE-conjugated Anti-SSEA-1	1:50	FACS	Miltenyibiotec	130-117-689	AB_2728020
