# Supplementary material for: Reconstituted ovaries self-assemble without an ovarian surface epithelium
Source: Stem Cell Reports. 2023 Oct 26;18(11):2190–202. doi: 10.1016/j.stemcr.2023.10.001 (PMC10679655; doi:10.1016/j.stemcr.2023.10.001)
Supplement: Document S2. Article plus supplemental information [file mmc2.pdf]

Stem Cell Reports
Article
OPEN ACCESS

Reconstituted ovaries self-assemble without an ovarian surface epithelium

Enrique Sosa,[1,2,3,4] Sinthia Kabir Mumu,[1,2,3,4] Christian C. Alvarado,[1,2,3,4] Qiu Ya Wu,[1,2,3,4] Isaias Roberson,[1,2,3,4] Alejandro Espinoza,[1,5] Fei-man Hsu,[1,2] Kaori Saito,[1] Timothy J. Hunt,[1] Jared E. Faith,[1] Matthew G. Lowe,[1,2,3] Jonathan A. DiRusso,[1,2,3,4] and Amander T. Clark[1,2,3,4,*]
[1]Department of Molecular, Cell and Developmental Biology, University of California, Los Angeles, Los Angeles, CA 90095, USA
[2]Eli and Edythe Broad Center of Regenerative Medicine and Stem Cell Research, University of California, Los Angeles, Los Angeles, CA 90095, USA
[3]Molecular Biology Institute, University of California, Los Angeles, Los Angeles, CA 90095, USA
[4]Center for Reproductive Science, Health and Education, University of California, Los Angeles, Los Angeles, CA 90095, USA
[5]Institute for Quantitative and Computational Biosciences – The Collaboratory, University of California, Los Angeles, Los Angeles, CA 90095, USA
*Correspondence: clarka@ucla.edu
https://doi.org/10.1016/j.stemcr.2023.10.001

SUMMARY

Three-dimensional (3D) stem cell models of the ovary have the potential to benefit women's reproductive health research. One such model, the reconstituted ovary (rOvary) self-assembles with pluripotent stem cell-derived germ cells creating a 3D ovarian mimic competent to support the differentiation of functional oocytes inside follicles. In this study, we evaluated the cellular composition of the rOvary revealing the capacity to generate multiple follicles surrounded by NR2F2+ stroma cells. However, the rOvary does not develop a surface epithelium, the source of second-wave pre-granulosa cells, or steroidogenic theca. Therefore, the rOvary models represent the self-assembly of activated follicles in a pre-pubertal ovary poised but not yet competent for hormone production.

INTRODUCTION

The ability to generate three-dimensional (3D) mammalian models of organogenesis in the lab have transformed biomedical research due to their capacity to achieve sufficient cellular complexity to represent key aspects of *in vivo* tissue morphogenesis outside the body. Most 3D cell and tissue models of the ovary have focused on the growth and maturation of postnatal or adult ovarian follicles, the functional units of the ovary (Cortvrindt et al., 1996; Laronda et al., 2017; Pangas et al., 2003; Xiao et al., 2017). In contrast, the reconstituted ovary (rOvary), generated through self-assembly of pluripotent stem cell (PSC)-derived germ cells with a single-cell suspension of embryonic ovarian somatic cells represents a complete model of ovarian organogenesis, including the *in vitro* specification of germline cells through to the self-assembly of ovarian follicles competent for *in vitro* fertilization (Hikabe et al., 2016). Given this accessibility, the rOvary technology holds exceptional promise as a screening platform to discover, test, and advance therapeutics that improve family planning and reproductive health. Therefore, it is imperative that the biology of the rOvary be well characterized.

The mammalian ovary derives from two distinct embryological sources. The germline, which originates from embryonic progenitors called primordial germ cells (PGCs) and the ovarian somatic support cells, which originate from bilateral genital ridges. In the mouse embryo, PGCs are specified between E6.25 and E7.5 from the posterior epiblast (Saitou et al., 2002). Specified PGCs initially settle in an extraembryonic structure called the allantois before migrating into the developing embryo toward the genital ridges (Ginsburg et al., 1990). Genital ridges develop from a single layer of GATA4+ coelomic epithelium on the ventro-medial surface of each mesonephros between E9.5 and E10.5 (Brennan and Capel, 2004; Hu et al., 2013). Once PGCs enter the genital ridges at around E10.5, they initiate expression of the PGC determination gene *Dazl* (Nicholls et al., 2019) and proliferate as germline cysts (Pepling and Spradling, 1998). The formation of germline cysts occurs within developing epithelial niches called ovigerous cords (Merchant, 1975), which originate from the GATA4+ ovarian epithelium. Specification of FOXL2+ pre-granulosa cells within the ovigerous cords is initiated from E11.5 (Mork et al., 2012; Schmidt et al., 2004; Wilhelm et al., 2009). Between E12.5 and E14.5 the germline cyst cells respond to retinoic acid and the germ cells enter prophase I of meiosis I, arresting at this stage until birth (Bowles et al., 2006).

At birth the ovigerous cords, consisting of pre-granulosa cells and meiotic oocytes, breaks down, leading to the formation of ovarian follicles (Pepling and Spradling, 2001). Each follicle is composed of a single germinal vesicle (GV) oocyte surrounded by a layer of FOXL2+ granulosa cells (Schmidt et al., 2004). Some ovarian follicles immediately activate, entering folliculogenesis (Mork et al., 2012) recruiting theca cells from the stroma (Barsoum and Yao, 2011). These are called first-wave follicles (Hirshfield, 1992). However, most follicles remain dormant creating the ovarian reserve. Analysis of rOvaries indicates a failure to establish dormant follicles (Shimamoto et al., 2019). Therefore, it can be hypothesized that the rOvary model represents the prepubescent first-wave of activated folliculogenesis.

Generation of rOvaries involves differentiating PGC-like cells (PGCLCs) from PSCs and combining them with either

This is an open access article under the CC BY license (http://creativecommons.org/licenses/by/4.0/).

germ-cell depleted fetal ovarian somatic cells (FOSCs) isolated from E12.5 embryonic ovaries (Hayashi et al., 2012; Hikabe et al., 2016), or *in vitro* differentiated FOS-like cells (FOSLCs) (Yoshino et al., 2021). The rOvary technology is exciting as it allows for reconstitution of the entire germline differentiation pathway including follicle formation entirely *in vitro*. Remarkably, the oocytes generated in this model are competent for *in vitro* maturation, fertilization and ultimately the birth of fertile offspring (Hikabe et al., 2016; Yoshino et al., 2021). However, to understand the potential biomedical uses of rOvaries in research, a thorough understanding of *in vitro* and *in vivo* ovarian morphogenesis is required.

In this study, we used the FOSC model for generating rOvaries to examine the cellular components before and after self-assembly of follicles. We focused on evaluating the ovarian surface epithelium, which generates the second wave-pre-granulosa cells (Mork et al., 2012), the stroma (Rastetter et al., 2014) and the theca cells (Newman Hirshfield, 1991). Here, we show that the ovarian surface epithelium is not present at the conclusion of rOvary formation, and although NR2F2+ stromal fibroblasts are located between the activated follicles, there is no recruitment of steroidogenic theca. Collectively this work suggests the rOvary represents a model for prepubescent ovary development and the premature activation of follicles.

RESULTS

Characterization of somatic and germ cells differentiated from iPSCs

Using a Blimp1-membrane bound Venus (BV), Stella-ECFP (SC) mouse induced PSC (iPSC) reporter line (Hikabe et al., 2016), we established a bank of karyotypically normal BVSC iPSCs for use in this project (Figure S1A). For each experiment, we thaw a single vial of iPSCs, passage once, and culture for 3 days before conversion into epiblast-like cells (EpiLCs) (Figures 1A and 1B). EpiLCs are harvested on day 2 of culture and plated as single cells in ultralow adhesion plates for 6 days with cytokines to promote 3D assembly with PGCLC and somatic cell differentiation (Figure 1B). To identify the major cell types on day 6 of differentiation as 3D assemblies, we performed single-cell RNA sequencing (scRNA-seq) using 10X Genomics from four independent differentiation experiments (Figure S1B; Table S1).

Using 10X Genomics at day 6 of differentiation, 5 major cell types in 10 clusters were consistently identified in each experiment (Figure S1B). This includes a major population of Tbx4+/Hand1+ mesoderm, a minor population of Kdr+/Tal1+ mesoderm, a putative *Tfap2c*+/*Hand1*+ trophoblast/amnion population and two populations with a pluripotency signature of *Oct4*, *Nanog*, and *Sox2* (Figures 1C and 1D). The most abundant mesoderm population expresses *Tbx4*, a transcription factor of the posterior allantoic mesoderm and vascular portions of the placenta (Inman and Downs, 2007). Suggesting that formation of the somatic niche co-differentiating with PGCLCs is similar to the mesoderm where PGCLCs initially locate after specification *in vivo*.

Analysis of the two clusters with pluripotency signatures reveals that one co-expresses the PGC specification signature of *Dppa3*, *Tfap2c*, *Prdm1*, and *Prdm14* (cluster 7, Figure 1D). We refer to this population as PGCLCs. In contrast, the other cluster (cluster 9) has limited expression of PGC signature genes (Figure 1D). We refer to cluster 9 as stem-like cells. In the PGCLC cluster (cluster 7), we also reliably detect transcription of a determination signature (*Dazl* and *Ddx4*), with no expression meiotic or oocyte genes including *Stra8*, *Zglp1*, and *Spo11* or *Figla* (Figure 2D). This result indicates that differentiation of germline *in vitro* occurs in a mesodermal niche, with evidence that some PGCLCs initiate PGC determination and therefore would correspond to E9.5–E10.5 PGCs (Figure 1D).

To confirm that STELLA (also called DPPA3) protein is transcribed from the *Dppa3* locus and is expressed by SC+ cells, we performed immunofluorescence (IF) at day 6 using cryosections that retain fluorescence activity of the reporters. This result reveals that STELLA protein is restricted to clusters of SC+ cells that also express BV (Figure 1E). We also identified some single BV+ cells that are negative for both STELLA and the SC transgene. Single BV+ cells have been reported previously in PGCLC differentiation experiments, and correspond to precursor PGCLCs that are yet to express STELLA protein (Hayashi et al., 2011). In summary, combining the results of 10X Genomics scRNA-seq with IF indicates that *Dppa3*/STELLA+ cells overlap, and these are enriched within the BV+ population.

scRNA-seq transcriptomic profiling of thawed FOSCs

For manufacturing of rOvaries, a reliable and consistent supply of FOSCs is required. The published technique for creating FOSCs involves collecting ovaries still connected to the mesonephros from the embryos of ten E12.5 time-mated pregnant females (Hayashi et al., 2012, 2017). Following removal of the mesonephros, and pooling and digestion of the ovaries with trypsin, a single-cell suspension of embryonic ovaries is created allowing for endogenous E12.5 PGCs to be depleted (but not eliminated) using magnetic-activated cell sorting (MACS) (Figure 2A). For off-the-shelf manufacturing of rOvaries, it is desirable to have a frozen stock (lot) of FOSCs made from the 10 time-mated pregnant females (Hayashi et al., 2017).

To identify the major embryonic ovarian cell populations in the FOSC lots after thaw, we performed 10X

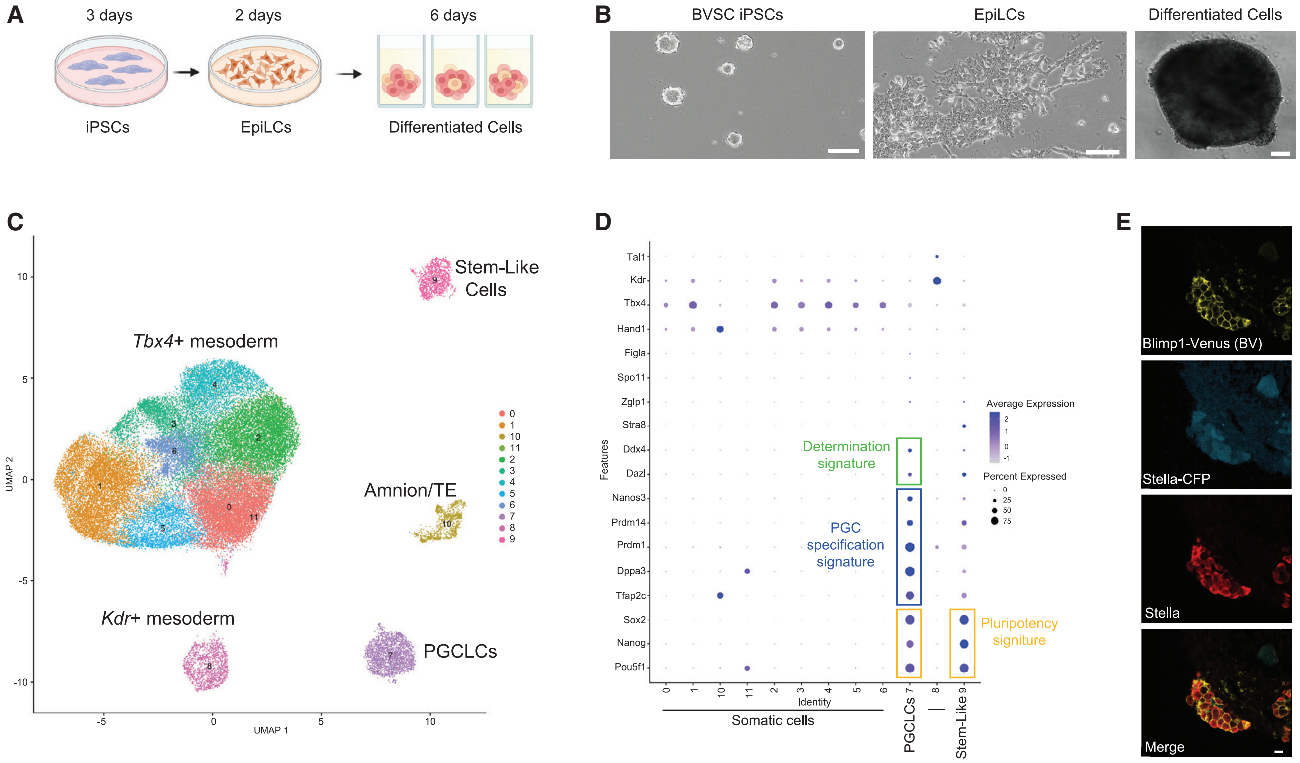

Figure 1. PGCLCs are induced with mesoderm during *in vitro* differentiation

(A) Schematic showing steps of PGCLC and somatic cell differentiation *in vitro*. Created with biorender.com.

(B) Representative phase contrast image of iPSCs, epiblast-like cells (EpiLCs), and a 3D assembly of day 6 differentiated cells. Scale bar, 100 μm.

(C) UMAP of cell types at day 6 of differentiation reveals five major populations corresponding to 10 clusters. Cluster 7 corresponds to PGCLCs based on the signature in (D). TE, trophoblast. A total of 45,192 validated cells were analyzed in this experiment (n = 4 independent differentiation experiments).

(D) Gene expression signatures in the 10 clusters shows the majority of somatic cells express the gene *Tbx4*.

(E) Immunofluorescence (IF) of frozen sections to evaluate STELLA protein. Venus (BV; yellow) and cyan fluorescent protein (SC; blue) cells correspond to BVSC PGCLCs. The BV transgene corresponds to a membrane-bound fluorescent protein. Notably BVSC PGCLCs are identified in clusters at day 6. Scale bar, 10 μm. See also Figure S1 and Table S1.

Genomics on three randomly selected lots (Figure 2B; Table S2). A small population of contaminating Sertoli cells was identified in all lots (0.1%–3.3%) (Figure 2C, cluster 10; Table S3) which were not analyzed further. Analysis of the embryonic ovarian cells in each lot revealed a similar identity following freeze-thaw (Figures 2C and 2D). The major ovarian cell types in the thawed FOSCs include *Wnt4/Foxl2*+ pre-granulosa cells, *UpK3B/Krt19/Nr2f2* epithelial cells, and *Nr2F2+/Tcf21*+ stromal fibroblasts cells (Figure 2D). A small population of *Ddx4/Dazl*+ PGCs can still be identified corresponding to 7.40%, 3.90%, and 3.41% in lot 26, lot 29, and lot 30, respectively, as well as minor populations of endothelial cells, macrophages, and red blood cells (Figure 2D; Table S3). Cell viability before and after MACS to create the FOSCs was >90% (Figure 3A). To confirm identity of MACS-escaped germ cells, we thawed three additional lots of FOSCs and performed flow cytometry for SSEA1 (Figure 3B). This result demonstrates that SSEA1+ MACS-escaped germ cells correspond to 5.13%, 2.88%, and 1.70% in lot 43, lot 47, and lot 58, respectively, in range with the predicted estimates by 10X Genomics.

Analysis of protein expression in aggregates of FOSCs via IF reveals randomly distributed VASA+ MACS-escaped germ cells interspersed near FOXL2+ pre-granulosa cells (Figure 3C). NR2F2+ stroma and KRT19 epithelial cells are also randomly organized at this stage, and laminin protein can be detected in the extracellular spaces between the cells (Figure 3C). Postnatal day 14 ovaries were used as a positive control for IF staining (Figure 3C). This result indicates that the production of frozen FOSCs is a viable strategy for creating a consistent off-the-shelf supply of cells for generating rOvaries. In addition, the presence of a laminin matrix at day 2 suggests frozen FOSCs are competent to initiate ECM deposition.

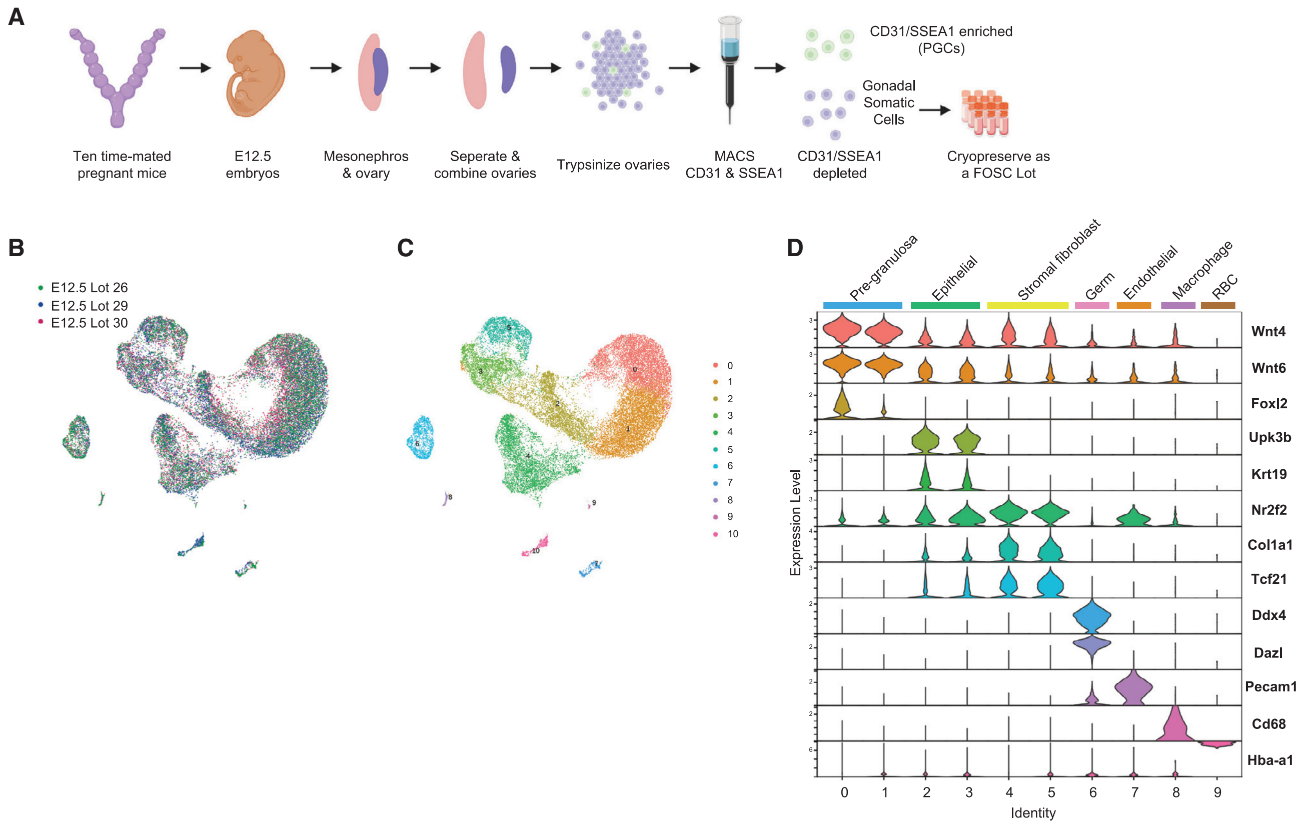

Figure 2. FOSCs are enriched in pre-granulosa cells, stromal fibroblasts, and epithelial cells

(A) Model for generating E12.5 FOSCs. A lot is generated by pooling a single-cell suspension of E12.5 ovaries isolated from the embryos of 10 time-mated pregnant CD1 mice. Germ cells are depleted from the single-cell suspension of ovaries using magnetic-activated cell sorting (MACS) prior to cryopreservation as a lot. Created with biorender.com.

(B) UMAP display of scRNA-seq 10X Genomics data from FOSCs immediately upon thaw of a cryopreserved lot revealing that lots consist of cells with similar identities (n = 3 independent lots for a total of 28,908 validated cells were analyzed). Cluster 10 corresponds to Sertoli cells and these were removed before characterization of ovarian cell types in (D).

(C) Data from (B) revealing the identity of 10 ovarian clusters (0–9).

(D) Violin plots of clusters 0–9 from (B) revealing that the FOSCs are composed mostly of pre-granulosa cells, epithelial cells, and stromal cells. The germ cells that escaped MACS are also detectable by DDX4. See also Tables S2 and S3.

Consistent manufacturing of rOvaries from frozen FOSCs

To generate rOvaries by *in vitro* differentiation (IVDi), we generated PGCLCs according to Figure 1, aggregated the PGCLCs with thawed vials of FOSCs in low-adhesion plates, and transferred the aggregates to the air-liquid interface for 21 days of IVDi to manufacture rOvaries (Hikabe et al., 2016) (Figure 4A). Differentiation of iPSCs to create PGCLCs at day 6 reveals a fluorescence signal corresponding to BV (representing mVenus protein expressed from *Blimp1* regulatory elements) overlapping with SC (representing CFP protein expressed from the *Stella* regulatory elements), particularly at the edges of each aggregate (Figure 4B), consistent with the location of PGCLCs identified using cryosections and IF (Figure 1E). FACS at day 6 reveals a BVSC double-positive PGCLC population (Figure 4C, box), and some single-positive BV cells (Figure 4C). Combining BVSC double-positive PGCLCs with thawed lots of FOSCs results in aggregates where TFAP2C+ PGCLCs can be identified dispersed randomly throughout the aggregate relative to FOXL2+ pre-granulosa cells (Figure S2A). We also observe bright DDX4+ MACS-escaped germ cells that do not express TFAP2C, consistent with the older developmental age of the endogenous PGCs compared with PGCLCs (Figure S2A).

Following 2 days (+2) as aggregates in 96-well plates, the aggregates are transferred to collagen membranes for 21 days of IVDi (Figure 4A). One day after transfer, SC+ PGCLCs can be identified, and are distributed throughout

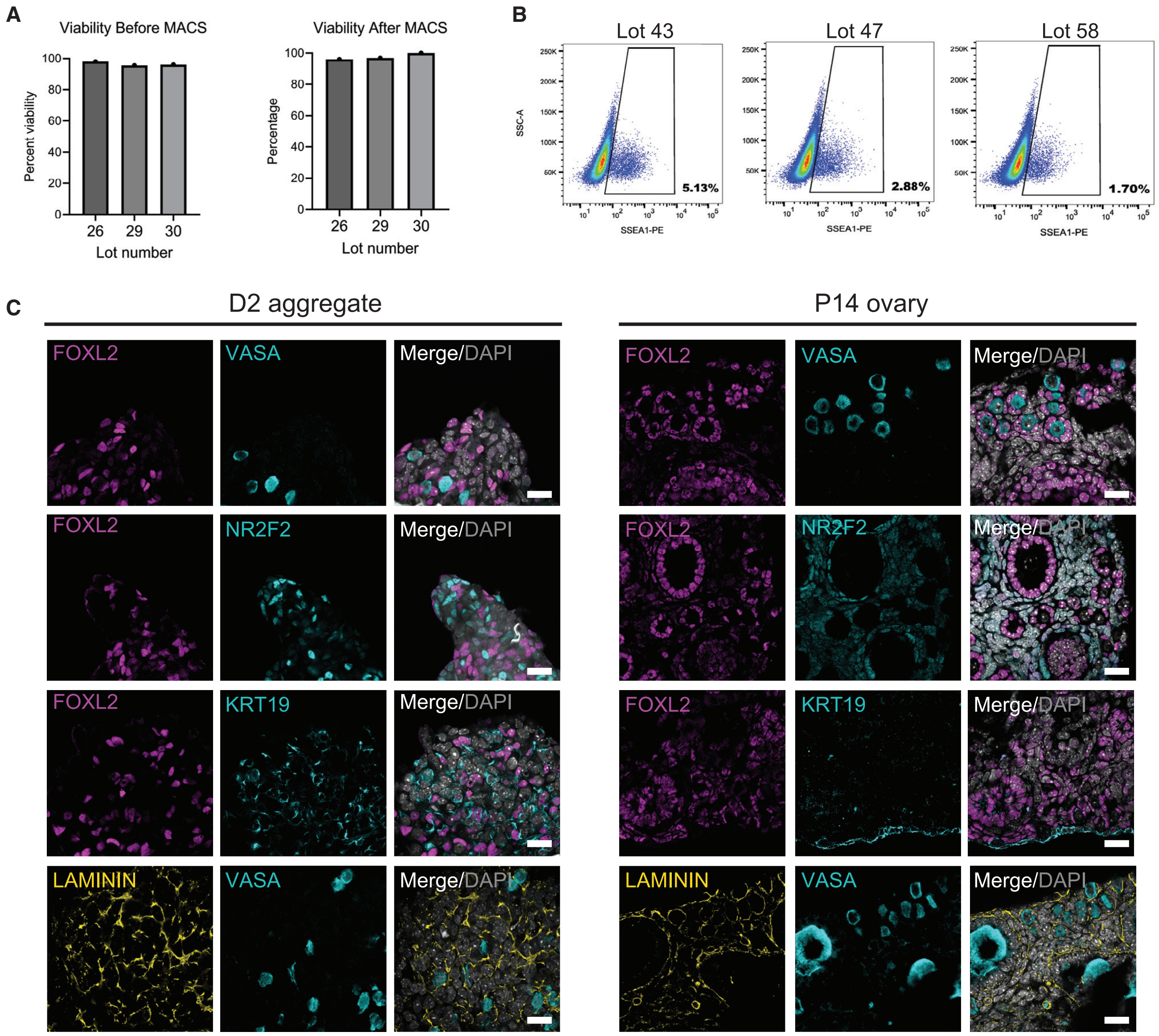

Figure 3. Thawed FOSCs are disorganized 2 days after aggregation

(A) Percent viability of the single-cell suspension of E12.5 ovarian cells before and after MACS prior to cryopreservation.

(B) Percentage of SSEA1-PE+ MACS-escaped germ cells following thaw of three different lots of FOSCs. SSC-A, side scatter; PE, phycoerythrin.

(C) IF of FOSCs aggregated for 2 days to evaluate major cell types in the aggregates (left). Postnatal day 14 (P14) ovary used as a positive control (right). Antibodies include FOXL2 (pre-granulosa/granulosa cell marker, pink), NR2F2 (ovarian stromal marker, blue), KRT19 (epithelial marker, blue), laminin (ECM, yellow), VASA (germ cell, blue). Scale bar, 20 μm.

the putative rOvary. By day 8, SC expression is reduced, and round balls of SC+ presumptive oocytes are starting to emerge. We hypothesize that the reduction in SC+ signal between days 1 and 8 may be due to fetal oocyte attrition and/or reduced levels of SC transgene as the germline enters meiosis (Ohinata et al., 2008). From days 15 to 21, individual SC+ oocytes can be distinguished within the rOvary (Figure 4D). To better understand how variability in FOSC lots might impact oocyte formation, we counted the number of SC+ oocytes in each rOvary and discovered no significant difference in the number of SC+ oocytes when comparing different lots (Figure 4E). We also did not observe any difference in the average size of SC+ oocytes (Figure 4F). We repeated this experiment with

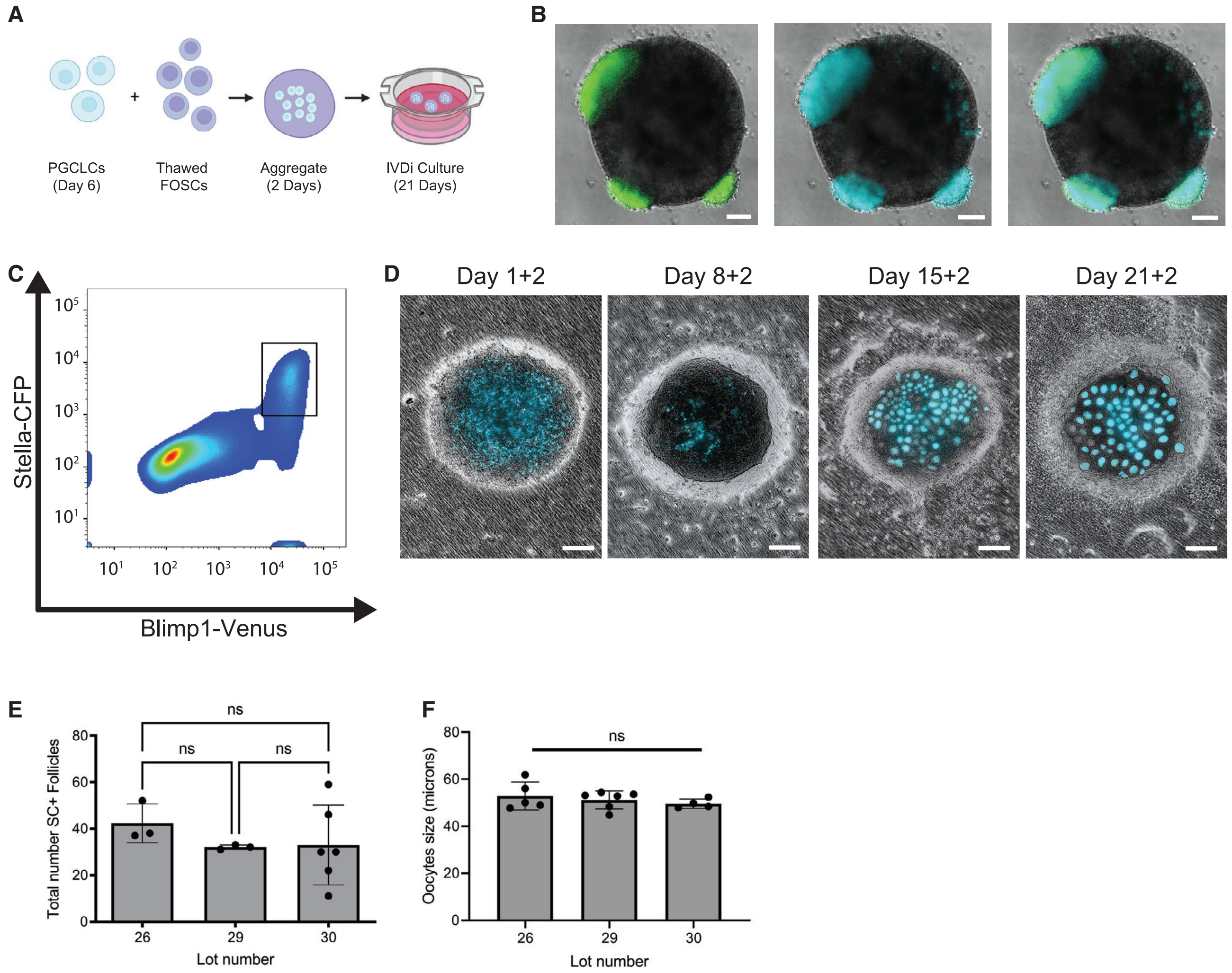

Figure 4. Organization of FOSCs and PGCLCs into follicles containing oocytes

(A) Model for generating rOvaries with PGCLCs and FOSCs. The first step involves 2 days of aggregation followed by 21 days of IVDi culture (23 days total). IVDi, *in vitro* differentiation. Created with biorender.com.

(B) Day 6 differentiated cells show BVSC+ PGCLCs at the edges of the 3D assemblies. Scale bar, 100 μm.

(C) FACS at day 6 for BV and SC. Box shows the double-positive BVSC PGCLCs.

(D) Representative phase contrast images overlaid with SC showing rOvaries at indicated days of IVDi. The annotation +2 refers to the 2 days of aggregate culture prior to transfer to the collagen membranes. Scale bar, 200 μm.

(E) Total number of SC+ oocytes at day 21 of IVDi from three different FOSC lots. Each dot represents the number of SC+ oocytes in a single rOvary. A minimum of n = 3 rOvaries were analyzed for this experiment. Data are shown as mean and standard error of the mean (SEM). Statistical analysis involved an ordinary one-way ANOVA followed by Tukey's multiple comparison test. Significance was accepted if $p < 0.05$.

(F) Oocyte size was measured at day 21. Each dot represents the average oocyte size in a single rOvary. Data are shown as mean and SEM. Statistical analysis involved an ordinary one-way ANOVA followed by Tukey's multiple comparison test. Significance was accepted if $p < 0.05$. See Figure S2.

additional frozen lots of FOSCs, and found a consistent and reproducible number and size of SC+ oocytes in our experiments (Figure S2B). IF for Stella at D21 shows that SC+ oocytes are positive for endogenous STELLA, indicating recapitulation of *in vitro* oogenesis from BVSC+ PGCLCs (Figure S2C). The average SC+ oocyte size is ~50 μm, and this is consistent with oocyte size during the first wave of activated follicles between postnatal days 12 (P12) and P17 (Chen et al., 2022). In addition to SC+ oocytes, SC– oocytes were also identified at D21 + 2, with the total number of

MACS-escaped (SC–) oocytes in each rOvary from each lot shown in Table S4.

Cellular assembly of rOvaries

Manipulation of rOvaries at the conclusion of IVDi has largely focused on breaking the rOvary into individual follicle units for *in vitro* growth, maturation, and fertilization (Hikabe et al., 2016). Given the potential utility of rOvary technology to study ovarian development, we examined the structure of the rOvary at day 21 of IVDi by embedding the rOvaries still attached to the collagen membrane in paraffin, and sectioning the rOvaries perpendicular to the membrane to visualize rOvary thickness (Figure S3A). This result shows that rOvaries are relatively flat disc-like structures about the width of a single follicle (Figure S3B). Using IF, we evaluated the major cell types of the D21 rOvary at the end of IVDi (Figures 5A–5E). Using a P14 ovary as a control (Figures 5F–5J), our data reveal that the follicles of rOvaries are composed of one to two layers of FOXL2+ pre-granulosa cells that surround a single DDX4+ oocyte (Figure 5A). Using NR2F2 to identify ovarian stromal fibroblasts, we stained FOXL2 together with NR2F2, and found that NR2F2+ cells are located between the follicles (Figures 5B and 5G), with some NR2F2+ cells found to co-express FOXL2. Using 3βHSD to mark steroidogenic theca recruitment from the stroma, we show that rOvaries do not have evidence of 3βHSD activity at the end of IVDi (Figure 5C), whereas the multi-layer follicles of P14 ovaries do (Figure 5H). Using laminin, we show a delicate basement membrane separates follicles from the stroma in the rOvaries (Figure 5D) and P14 ovaries (Figure 5I). The surface of the rOvaries did not have a distinct basal lamina, suggesting that this model may lack a surface epithelium. To confirm this, we stained the rOvaries (Figure 5E), and P14 ovaries (Figure 5J) with KRT19. The results clearly show a KRT19 ovarian surface epithelium on the ovary at P14, whereas only rare KRT19+ cells are identified in rOvaries, and these do not form a surface epithelium.

DISCUSSION

Here, we show that the rOvary technology is a reliable and reproducible approach to support the differentiation of PGCLCs into oocytes. In our hands, the average number of follicles generated in the rOvary model corresponds to 48 follicles containing SC+ oocytes, and 10 follicles containing SC– oocytes per rOvary. Based on oocyte size, the follicles containing SC+ oocytes generated at the conclusion of IVDi (23 days after assembly with FOSCs) correspond to activated follicles equivalent to those found in immature pre-pubertal postnatal ovaries. The lack of 3βHSD indicates that the activated follicles have not yet initiated recruitment of NR2F2+ stromal cells to become steroidogenic theca. Furthermore, the lack of KRT19 on the surface of rOvaries indicates that this model does not assemble an ovarian surface epithelium.

The presence of MACS-escaped germ cells in the FOSCs and MACS-escaped oocytes following IVDi has been reported previously (Hayashi et al., 2017; Hikabe et al., 2016). Given that we begin the experiment with 5,000 PGCLCs and acquire 48 SC+ oocytes on average in each rOvary, we calculate the average survival and differentiation of PGCLCs into oocytes as 1%. Early and late oocyte attrition are poorly understood processes that lead to death of the majority of the germline pool (Hunter, 2017). Therefore, the rOvary model will likely to provide valuable insights into this process in future studies.

The ovarian surface epithelium is the source of pre-granulosa cells found in quiescent follicles of the adult ovary (Mork et al., 2012; Ng et al., 2014; Niu and Spradling, 2020; Rastetter et al., 2014). The lack of ovarian surface epithelium (but presence of follicles) could be explained by the development ontogeny of granulosa cells, which involves two distinct waves. As GATA4+ cells of the coelomic epithelium proliferate to form the genital ridges, the underlying basement membrane breaks down, and the epithelial cells express transcription factors essential for bipotential genital ridge development including *Nr5a1* (Luo et al., 1995; Stévant et al., 2019), *Lhx9* (Birk et al., 2000), and *Emx2* (Kusaka et al., 2010). Sex determination begins soon after E10.5 with expression of the *Sry* mouse sex-determining gene, leading to the epithelial cells adopting a testis fate (Hacker et al., 1995), or β-catenin signaling by Rspo1 leading to an ovary fate (Chassot et al., 2008; Liu et al., 2009). In the embryonic ovary, *Foxl2* expression is initiated in nascent pre-granulosa cells starting around the time of sex determination at E11.5 (Mork et al., 2012; Schmidt et al., 2004; Wilhelm et al., 2009), which is 24 h before FOSC extraction at E12.5.

FOXL2+ pre-granulosa cells at E12.5 are called first-wave pre-granulosa cells (Mork et al., 2012). The second-wave pre-granulosa cells are specified from *Lgr5*+ precursors originating in the ovarian surface epithelium (Ng et al., 2014; Rastetter et al., 2014). These *Lgr5*+ second-wave precursors do not express FOXL2 protein until after birth (Niu and Spradling, 2020). Lineage tracing of *Foxl2*-expressing pre-granulosa cells at E12.5 and E14.5 reveals that FOXL2+ first-wave pre-granulosa cells between E12.5 and E14.5 contribute to ovarian follicles that undergo rapid and direct follicle activation (Mork et al., 2012). In contrast, the *Lgr5*-expressing pre-granulosa cells from the ovarian surface epithelium give rise to the future dormant ovarian cortical follicles from around E14.5 (Niu and Spradling, 2020; Rastetter et al., 2014). Given that FOSCs used in rOvary formation are isolated from E12.5, it can be hypothesized that the

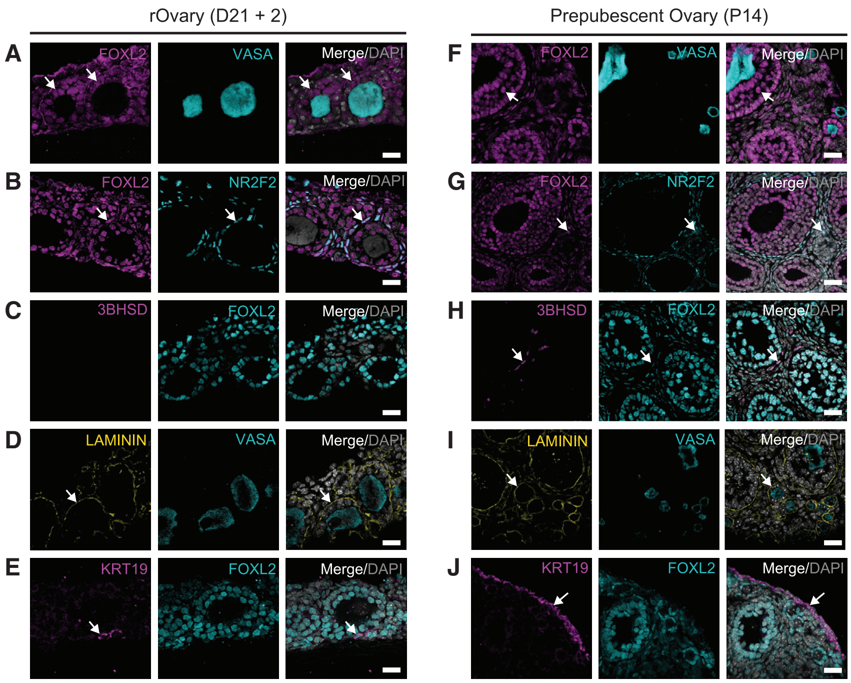

Figure 5. rOvaries do not have an ovarian surface epithelium or steroidogenic theca

(A) IF of rOvaries for FOXL2 (pre-granulosa/granulosa marker (pink) and VASA (germline, blue). Arrows point to follicles. All rOvary images (A–E) are at day 21 of IVDi. P14 (F–J) was used as a positive control. Scale bars, 20 μm (A–J).

(B) IF for FOXL2 (pink) and NR2F2 (stroma marker, blue) in day 21 of rOvaries. Arrows point to FOXL2+ NR2F2+ stromal cells.

(C) IF of 3βHSD (theca marker, pink) and FOXL2 (blue) in day 21 of rOvaries

(D) laminin (yellow) and VASA (blue) in day 21 rOvaries. Arrows point to basement membrane surrounding individual follicles.

(E) KRT19 (ovarian epithelial marker, pink) and FOXL2 (blue) in day 21 rOvaries. Arrows point to rare KRT+ cells in the rOvaries.

(F) IF of CD1 mouse ovaries at postnatal (P) day 14 FOXL2 (pre-granulosa/granulosa marker, pink) and VASA (germline, blue). All ovaries are from a P14 mouse. Arrow points to examples of follicles.

(G) FOXL2 (pink) and NR2F2 (stroma marker, blue) in P14 ovaries. Arrow points to FOXL2+ NR2F2+ cells in the stroma.

(H) 3βHSD (theca marker, pink) and FOXL2 (blue) in P14 rOvaries. Arrow points to steroidogenic theca cells.

(I) Laminin (yellow) and VASA (blue) in P14 rOvaries. Arrow points to basement membrane surrounding individual follicles.

(J) KRT19 (ovarian epithelial marker, pink) and FOXL2 (blue) in P14 rOvaries. Arrow points to the KRT+ surface epithelium of the postnatal ovary. See Figure S3 and Table S4.

resulting activated rOvaries originate from FOXL2+ first-wave pre-granulosa cells.

Not only does the presence of FOXL2+ pre-granulosa cells in day 2 aggregates support the hypothesis that the follicles of rOvaries originate from the first-wave pre-granulosa cells, so does the size of the GV-stage oocytes, which corresponds to the size of the first-wave oocytes in primary/early secondary follicles (Chen et al., 2022). Recent studies of FOXO3 in the rOvary model reveals that this protein localizes to the oocyte cytoplasm at day 11 of IVDi when follicles are first formed (Hikabe et al., 2016; Shimamoto et al., 2019). This is critical as cytoplasmic FOXO3 is associated with follicle activation (John et al., 2008). It is possible that some follicles in the rOvaries may contain second-wave pre-granulosa cells given that rare *Lgr5*-expressing cells are detected in the embryonic ovary from E11.5 (Mork et al., 2012; Ng et al., 2014; Niu and Spradling, 2020; Rastetter et al., 2014). However, further investigation of this is needed.

Although NR2F2+ ovarian stroma is detected between the follicles at day 21 of IVDi, there is no evidence of steroidogenic activity in these cells, which would be indicative of theca recruitment. In contrast, the P14 ovaries exhibit 3βHSD expression in the stromal cells around the larger multi-layer follicles. The absence of steroidogenic theca cells in the ovaries at day 21 of IVDi is not surprising given that the follicles in the rOvary correspond to primary early secondary follicles, whereas theca recruitment begins in multi-layer pre-antral follicles downstream of GDF9 secretion from the growing oocyte (Dong et al., 1996). In the rOvaroid model where FOSLCs are generated from ESCs rather than isolated from embryos, Nr5a1-hCD271 (a theca marker) can be identified around activated follicles (Yoshino et al., 2021). However, it was not reported whether these theca cells are steroidogenic or not. Theca cells in the ovary originate from two sources, the WT1 cells from the ovary medulla and the Gli1+ mesenchymal cells that migrate in from the mesonephros (Liu et al., 2015). As part of FOSC generation, the mesonephros is removed, therefore, it is likely that the Gli1+ mesonephric theca progenitors are also removed. We speculate that this should not be a major issue as WT1 progenitors are the dominant source of theca in the adult ovary, and these cells are identified in the developing embryonic ovary as early as E11.5 (Liu et al., 2015), which is prior to FOSC extraction. Therefore, if steroidogenic theca cells can be produced with

additional rOvary culture, it is likely that these will originate from WT1 progenitors.

In summary, we have demonstrated that self-assembly of rOvaries mimics key aspects of ovarian biology including the generation of GV-stage oocytes with one to two layers of granulosa cells. At the conclusion of IVDi, the model is poised to enter the gonadotrophic-dependent phase and produce ovarian hormones, an activity that does not require surface epithelium. Hormone production by the rOvary will require the model to be competent to generate steroidogenic theca.

EXPERIMENTAL PROCEDURES

Resource availability

Corresponding author

Further information and requests for resources and reagents should be directed to and will be fulfilled by the lead contact, Dr Amander Clark (clarka@ucla.edu). Requests for the BVSC iPSC line used in these studies should be made to hayashik@gcb.med.osaka-u.ac.jp.

Materials availability

This study did not generate new unique reagents.

Data and code availability

The accession numbers for the sequencing data reported in this paper are GEO: GSE238263 and GEO: GSE239488. Any additional information will be available from the corresponding author upon request.

Experimental model and subject details

Mouse iPSCs

The transgenic BV and SC mouse iPSC reporter lines (clone 4 FR C3) was published previously (Hikabe et al., 2016). The mouse iPSC line used for these studies was derived from tail tip fibroblasts of a 10-week-old adult female mouse. The female donor mouse was generated by crossing a 129X1/SvJ (chinchilla) female and C57BL/6 BVSC male.

Animals

FOSCs were prepared from embyros of CD1 pregnant mice (Charles River, strain code: 022).

Method details

PSC culture

BVSC iPSCs were cultured in six-well tissue culture plates coated with 0.01% poly-L-ornithine solution (Sigma, catalog no. P4957-50ML) and laminin at 10 ng/mL (Sigma, catalog no. L2020). BVSC iPSCs were thawed in 2i+LIF medium (N2 supplement [Gibco, catalog no. 17502-048], B27 supplement [Gibco, catalog no. 12587-010], DMEM/F12 w/o HEPES [Gibco, catalog no. 11320-033], neurobasal medium (Gibco, catalog no. 11360-070), 0.5 μM PD0325901 inhibitor (Stemgent, catalog no. 04–0006), 3 μM CHIR99021 inhibitor (Reprocell, catalog no. 04-0004-10), 1× β-mercaptoethanol (Gibco, catalog no, 21985-023), penicillin-streptomycin-glutamine (Gibco, catalog no. 10378-016), and 10,000 U/mL ESGRO mouse LIF (Millipore, catalog no. ESG1107), and cultured for 3 days at 37°C, 5.0% CO_2, with daily medium changes. On day 3, iPSCs were passaged using 0.05% trypsin-EDTA (Gibco, catalog no. 25300120) and re-plated at a concentration of 5 × 10^4 cells/well onto new poly-L-ornithine- and laminin-coated plates containing 2i+LIF medium. iPSCs were cultured for another 3 days receiving daily medium changes before generating EpiLCs.

EpiLC induction

After 3 days of culture, BVSC iPSCs were dissociated into a single-cell suspension using 0.05% trypsin-EDTA (Gibco, catalog no. 25300120). EpiLCs were induced by plating 1.0 × 10^5 cells/well of 12-well plates (Corning, catalog no. 3513) coated with 16.7 μg/mL human plasma fibronectin (Invitrogen, catalog no. 33016-015), and cultured in EpiLC medium. EpiLC medium is composed of 1× N2 supplement (Gibco, catalog no. 17502-048) diluted in DMEM/F12 w/o HEPES (Gibco, catalog no. 11320-033), 1× B27 supplement (Gibco, catalog no. 12587-010) diluted in neurobasal medium (Gibco, catalog no.11360-070) (N2B27). To the N2B27 medium the following supplements are added, 20 ng/mL Activin A (Peprotech, catalog no. AF-120-14E), 12 ng/mL HumanKine recombinant FGFbasic-TS protein (Proteintech, catalog no. HZ-1285), 1% KSR (Gibco, catalog no. 10828-028), 1× β-mercaptoethanol (Gibco, no. 21985-023), and 1× penicillin-streptomycin-glutamine (Gibco, catalog no. 10378-016). The cells are cultured at 37°C, 5.0% CO_2 for 2 days to generate EpiLCs, with a fresh medium change on day 1. DMEM/F12 with HEPES has been reported to cause non-specific upregulation of the BV transgene (Hayashi and Saitou 2013). Therefore DMEM/F12 without HEPES is required (Hayashi and Saitou, 2013). Preparation of N2 stock in DMEM/F12 without HEPES solution must be made fresh prior to preparing N2B27 in neurobasal medium. N2B27 is required for the differentiation of EpiLCs from 2i+LIF-cultured PSCs (Hayashi and Saitou, 2013).

Primordial germ cell-like cell and somatic cell differentiation

After 2 days of EpiLC culture, cells were dissociated into a single-cell suspension using 0.05% trypsin. PGCLCs were induced in 3D by seeding 2.0 × 10^3 cells into each well of a 96-well ultra-low attachment multiwell plate (Corning, catalog no. 7007) in GK15 medium (GMEM [Gibco, catalog no. 11710-035], 15% KSR [Gibco, catalog no. 10828-028), 1× NEAA [Gibco, catalog no. 11140-050], 1× sodium pyruvate [Gibco, catalog no. 11360-070], 1× β-mercaptoethanol [Gibco catalog no. 21985-023], and penicillin-streptomycin-glutamine [Gibco, catalog no. 10378-016]) with the addition of the following cytokines: 500 ng/mL mouse BMP-4 recombinant protein (R&D Systems, catalog no. 5020BP010/CF), 500 ng/mL recombinant mouse BMP-8a protein (R&D Systems, catalog no. 7540-BP-025), 50 ng/mL recombinant mouse EGF protein CF (R&D Systems, catalog no. 2028-EG-200), 100 ng/mL stem cell factor recombinant mouse (Peprotech, catalog no. 250-03), and 10,000 U/mL ESGRO mouse LIF (Millipore, catalog no. ESG1107). Differentiation ultra-low attachment multiwell plates was performed for a total of 6 days at 37°C, 5.0% without any medium changes.

Fluorescence-activated cell sorting

On day 6 of PGCLC differentiation, cells were collected and dissociated using 0.05% trypsin-EDTA (Gibco, catalog no. 25300120) for 10 min at 37°C. The cell suspension was washed with MEF medium (high-glucose DMEM [Invitrogen, catalog no. 11960-069], 10% FBS

[Invitrogen, catalog no. 26140-079], 1× penicillin-streptomycin-glutamine, and 1× Primocin (Invivogen, catalog no. ant-pm-2]) by centrifugation at 1.2 rpm for 5 min at room temperature. Cells were then resuspended in FACS buffer (1% BSA [Sigma, catalog no. A3311-100G] and 1× PBS]) and transferred to a Falcon 12 × 75-mm tube with a cell strainer cap (Corning, catalog no. 352235). Compensation controls were prepared from mouse iPSCs (without transgenes), Oct4-GFP iPSC reporter line, and a CFP-expressing iPSC cell line, which were plated 24 h before sorting. The cell viability dye 7-AAD (BD Biosciences, catalog no. 559925) was added (1:60 concentration) to the 7-AAD control and the experimental sample 15 min before FACS. BVSC double-positive PGCLCs were sorted using a BD FACSAria II cell sorter and collected in GK15 medium supplemented with 30 mg/mL retinoic acid.

MACS-escaped germ cell quantification by flow cytometry

Cryopreserved lots of FOSCs were thawed at room temperature and incubated with anti-human/mouse SSEA1 antibody (Miltenyi Biotec, catalog no. 130-117-689) (Table S5) in FACS buffer on ice at 1:50 for 15 min. After antibody incubation, the cells were washed twice in FACS buffer. 7AAD (BD Biosciences, catalog no. 559925) was added at 1:60 before analysis on a BD FACSAria II using BD FACSDiva 8.0.2 followed by post analysis processing in FlowJo 10.6.2 (BD Biosciences).

FOSC lot production and cryopreservation

FOSCs in each lot were obtained from 10 time-mated E12.5 pregnant female CD1 mice (Charles River, strain code: 022). Gonads, which included the mesonephros and ovaries, were collected into cell culture plates filled with MEF medium on ice. Gonads were transferred into glass plates containing MEF medium and mesonephros were removed using surgical blades and fine forceps under a SZX16 (Olympus) microscope. Ovaries were collected into a well of a four-well plate containing MEF medium and DNase I. Ovaries were washed twice by transferring them into wells containing PBS. Dissociation of ovaries was performed in 0.05% trypsin for 10 min at 37°C. MEF medium was used to quench and tissues were dissociated by vigorous pipetting. Cells were strained using a 70-μM cell strainer and then washed using MEF medium. The ovarian somatic cell pellet was then resuspended in MACS buffer composed of PBS (Invitrogen, catalog no. 11960-069) containing 0.1% BSA (Invitrogen, catalog no. A3311-100G) and 0.5 M EDTA (Thermo Fisher Scientific catalog no. AM9260G), and incubated with the MACS antibodies SSEA1 (Miltenyi catalog no. 130-094-530) and CD31 (Miltenyi catalog no. 130-097-418) at 4°C for 30 min (Table S5). Afterward, labeled cells were washed by adding MACS buffer before spinning for 5 min at 1,600 rpm. The cell pellet was resuspended in 500 μL MACS buffer and then loaded into an MS column held by a MiniMACS Separator magnet. Flowthrough was collected, including the MACS medium added for washes. The number of cells collected was counted and aliquots of 300,000 cells per vial were frozen down in Cell Banker 2 (AMSBIO, catalog no. 11914) at −80°C and then transferred to liquid nitrogen tanks for long-term storage. All animal research is conducted under regulatory oversight by the UCLA Office of Research. The work was conducted following review and approval by the UCLA Institutional Animal Research Oversight Committee, protocol no. ARC-2020-171.

Mouse rOvary generation

To generate rOvaries we combined 5.0 × 10^3 BVSC double-positive PGCLCs with 5.0 × 10^4 E12.5 FOSCs per well of a 96-well plate (Corning, catalog no. 7007) in GK15+ RA medium. Afterward, we centrifuged the 96-well plates at 200 rpm for 4 min (Beckman Coulter, Allegra X-15R centrifuge) and allowed the cells to aggregate for 2 days in an incubator at 37°C, 5.0% CO_2. On day 2 of aggregation, we transferred three to five aggregates onto COL-membrane supports (Sigma-Aldrich, catalog no. CLS3491-24EA) placed in six-well plates (Corning, catalog no. 3516) containing α-MEM-based IVDi medium (α-MEM (Invitrogen, catalog no. 12571-063), 2% FBS (LifeTech, catalog no. 26140079), GlutaMAX (Invitrogen, catalog no. 35050-061), β-mercaptoethanol, 1× penicillin-streptomycin (Invitrogen, catalog no. 15070), 0.15 mM ascorbic acid (Sigma-Aldrich, catalog no. A8960-5G)) using wide-orifice pipette tips (VWR, catalog no. 46620-642). Transfer of aggregates onto COL-membrane supports constituted day 0 of rOvary culture on an air-liquid interface. On day 2 of rOvary culture, α-MEM-based IVDi medium was changed. On days 4 and 6 of rOvary culture half medium changes were performed using StemPro-34-based IVDi medium (StemPro-34 basal medium with supplement [Gibco, catalog no. 10639-011], 10% FBS [LifeTech, catalog no. 26140079], 1× GlutaMAX (Invitrogen, catalog no. 35050-061), β-mercaptoethanol, 1× penicillin-streptomycin [Gibco, catalog no. 15070], 0.15 mM ascorbic acid [Sigma-Aldrich, catalog no. A8960-5G]). On day 7 of rOvary culture 12.14 mg/mL ICI182,780 (Tocris, catalog no. 1047), an inhibitor of estrogen signaling, was added to the medium. On day 8 of rOvary culture a half medium change was done using StemPro-34-based IVDi medium. On day 9 of rOvary culture, a full medium change with StemPro-34 IVDi and ICI182,780 was performed. On days 11, 13, 15, 17, and 19 of rOvary culture full medium changes were carried out using StemPro-34-based IVDi medium. On day 21 of culture, rOvaries were collected for downstream analysis.

Follicle counting and oocyte diameter measurements

On day 21 of rOvary culture, bright-field (BF) and CFP images were taken using a 10× objective for each individual rOvary and saved as individual oir files (Olympus CK×53). For each rOvary, BF and CFP channels were merged using Imaris 8.3.1 (Bitplane). Oocyte diameters were collected using the measuring tool within slice mode for all SC+ follicles. MACS-escaped germ cells were defined as follicles containing oocytes that were negative for SC. Criteria for selection of SC+ oocytes involved the SC signal being morphologically ovoid in shape and within a zone pellucida. Criteria for selecting SC– oocytes, the zona pellucida, and/or GV was visible and the shape of the oocyte was ovoid. No minimum size was excluded. To calculate statistical significance, an ordinary one-way ANOVA followed by Tukey's multiple comparison test was conducted using the GraphPad Prism v.10.0.0. Significance was accepted if $p < 0.05$.

Cryosectioning and IF

rOvaries on COL membranes were washed with PBS (1×; Fisher BioReagents, catalog no. BP3994) then fixed in 2% PFA on ice for 3 h. Samples were washed three times in PBS with 0.2% Tween (Fisher Scientific, catalog no. BP337-100) for 10 min each on ice. Next, rOvaries were placed in 10% sucrose solution (Sigma-Aldrich, catalog no. S0389) for 10 min on ice then transferred to a 30% sucrose solution and allowed to incubate overnight at 4°C.

Each rOvary was placed in Tissue-Tek O.C.T. Compound (Sakura, catalog no. 4583) and then snap-frozen using liquid nitrogen. Snap-frozen samples were sectioned at a thickness of 5 μm and placed onto glass slides (Fisherbrand, catalog no. 1255015). Slides were stored at −80°C. On day of IF staining slides were warmed to room temperature, then rinsed with PBS. Slides were then washed three times for 10 min each wash in 0.2% PBST. Blocking was performed at room temperature for 1 h using 10% normal donkey serum (Fisher Scientific, catalog no. NC9624464). Primary antibodies were incubated overnight at 4°C and then washed 6 times for 15 min each with PBS. Secondaries were added and incubated in the dark for 1 h at room temperature and then washed with PBS four times for 15 min each wash. Primary and secondary antibody concentrations are listed in Table S5. Mounting medium was added and coverslips were placed on slides and then sealed.

Paraffin processing and IF

Samples were washed with PBS then fixed in 2% PFA on ice for 3 h. Afterward, samples were washed with PBS and 0.2% Tween (0.2% PBST). rOvaries were then cut out from the membrane and washed three times with 0.2% PBST for 10 min each on ice and processed to paraffin. To prepare samples for IF staining, 5-μm slides were deparaffinized and rehydrated to PBS wash. Antigen retrieval was done in Tris-EDTA buffer at 95°C for 40 min. After a 20-min cool down at room temperature, slides were washed in PBS (1×) for 5 min. Samples were then placed in permeabilization buffer (PBS and 0.05% Triton X) for 20 min then washed three times for 5 min each wash in 0.2% PBST. Blocking was performed using 10% normal donkey serum at room temperature for 30 min. Primary antibodies were incubated overnight in 4°C and then washed three times for 5 min each with 0.2% PBST. Secondaries were added and incubated in the dark for 1 h at room temperature and washed with 0.2% PBST three times for 5 min each wash. Primary and secondary antibody concentrations are listed in Table S5. Sections were washed once with PBS for 5 min and DAPI (Fisher Scientific, catalog no. D1306) was applied for a maximum of 5 min. Sections were washed with PBS three times for 5 min each wash. Mounting medium was applied and a coverslip was added then sealed. The slides were allowed to cure overnight at room temperature in the dark.

Microscopy

Images were taken on a confocal laser scanning microscope (LSM880 or LSM780) (Carl Zeiss) using either a Plan-Apochromat 20×/0.8 NA objective or a Plan-Apochromat 63×/1.4 NA M27 oil immersion objective at room temperature. Acquired images were processed in the image processing software Imaris 8.3.1 (Bitplane).

Preparing cells for scRNA-seq by 10X genomics

Single-cell suspensions were prepared of the day 6 aggregates (n = 4 samples) and the thawed FOSC lots (n = 3 samples) using trypsin EDTA (Gibco, catalog no. 25300-054). Single cells were washed three to five times in cold PBS supplemented with 0.04% BSA. Single-cell suspensions were processed to 10X Genomics GEM formation and library preparation using the chromium single-cell 3′ Reagent Kits v.3 (10X Genomics, catalog no. PN-100075).

scRNA-seq analysis (FOSC lots)

Valid cells and UMIs were determined by UMI-tools (Smith et al., 2017) to generate the whitelist. Reads corresponding to valid barcodes were aligned to GRCm38 with STAR 2.7 (Dobin et al., 2013), and only uniquely mapped reads were kept for downstream analyses. Count matrices were generated by featureCounts v.2.0.1 from the Subread R package (Liao et al., 2013), with UMI information further appended to the alignment.bam file. The cell-by-gene count matrix was generated with umi_tools count function and served as input data for Seurat (Stuart et al., 2019). We only kept the cells expressing at least 200 genes, and genes with expression in at least 3 cells. The cells were also filtered by the maximum of 9,000 expressed genes and of 10% mitochondrial genes. The UMI counts were then normalized for each cell by the total expression, multiplied by 10,000, and log-transformed. We used Seurat's default method to identify highly variable genes and scaled data for regressing out variations. The scaled data with variable genes were used to perform principal-component analysis (PCA). The top 50 PCs were chosen for further analysis, including clustering to identify cell populations. Rare cells with testis identity were not included in the analysis. Uniform Manifold Approximation and Projections (UMAPs) were calculated by RunUMAP function in Seurat package using the top 50 PCs.

scRNA-seq analysis of PGCLCs and somatic cells

The raw GEX libraries were mapped to the mouse reference genome (refdata-gex-mm10-2020A) using cellranger count (v.6.1.2) from 10X Genomics. The aligned data were filtered using SoupX default parameters and the output was analyzed using the R package Seurat (v.4.0). Cells with more than 10% mitochondrial expression and more than 7,800, 12,300, 7,500, and 6,000 unique features were removed for aggregates 1, 2, 3, and 4, respectively. UMI counts were log normalized using NormalizeData default parameters, FindVariableFeatures to select the 2,000 highest variable genes, ScaleData, and PCA at 30 components. We then integrated the datasets using R package Harmony using default parameters, FindNeighbors, RunUMAP, and FindClusters at 30 components for UMAP visualization. We manually annotated germ and somatic cells using a curated set of gene markers.

Karyotyping

Routine G-band karyotyping was performed on BVSC iPSCs by Cell Line Genetics (Madison, WI). In brief, 4.0×10^5 to 6.0×10^5 BVSCs were plated onto 0.1% gelatin-coated T25 flask (Falcon, catalog no. 353082) and cultured for 2–3 days (until 80% confluent) at 37°C, 5.0% CO_2 in mESC medium (KnockOut DMEM [Invitrogen catalog no.10829-018] 15% FBS [Thermo Fisher Scientific, catalog no. SH3007003] 1× penicillin-streptomycin-glutamine [Gibco, catalog no. 10378-016], 1× non-essential amino acids [Invitrogen, catalog no.11140-050], 55 mM β-mercaptoethanol [Gibco, catalog no. 21985-023], 10,000 U/mL ESGRO [Milipore, catalog no. ESG1107]) then shipped to Cell Line Genetics for analysis. A total of 20 metaphase spreads were investigated during analysis.

SUPPLEMENTAL INFORMATION

Supplemental information can be found online at https://doi.org/10.1016/j.stemcr.2023.10.001.

ACKNOWLEDGMENTS

This work was supported, in part, by the Bill & Melinda Gates Foundation (INV-003385). Under the grant conditions of the Foundation, a Creative Commons Attribution 4.0 Generic License has already been assigned to the author accepted manuscript version that might arise from this submission. The authors would

also like to thank Flow cytometry and Next Generation Sequencing cores at the UCLA Eli and Edythe Broad Center of Regenerative Medicine and Stem Cell Research Center (BSCRC) for help with cell sorting, sequencing, and imaging. Also, we would like to thank the Translational Pathology Core Laboratory for help with histology and the UCLA MCDB/BSCRC Imaging core for confocal microscopy. ATC is supported by grants from the NIH R01HD079546, R01HD098278, and R01HD058047. We would also like to thank the members of the Oral Contraceptive Discovery Initiative (OCDI), Professors Francesca Duncan (Northwestern and PI of the OCDI, Shuo Xiao (Rutgers), and Britt Goods (Dartmouth) for helpful discussions.

AUTHOR CONTRIBUTIONS

A.T.C. conceived and supervised the project. E.S., S.K.M., C.C.A., I.R., K.S., T.J.H., J.E.F., M.G.L., and J.A.D. performed the experiments. F.-M.H. and A.E. conducted all of the computational analyses with help from J.A.D. A.T.C. wrote the manuscript with input from E.S., S.K.M., C.C.A., F.-M.H., A.E., I.R., and J.A.D. and agreement from all authors.

DECLARATION OF INTERESTS

A.T.C. is an office holder of the ISSCR.

Received: July 28, 2023
Revised: October 1, 2023
Accepted: October 2, 2023
Published: October 26, 2023

REFERENCES

Barsoum, I., and Yao, H.H. (2011). Redundant and differential roles of transcription factors Gli1 and Gli2 in the development of mouse fetal Leydig cells. Biol. Reprod. *84*, 894–899. https://doi.org/10.1095/biolreprod.110.088997.

Birk, O.S., Casiano, D.E., Wassif, C.A., Cogliati, T., Zhao, L., Zhao, Y., Grinberg, A., Huang, S., Kreidberg, J.A., Parker, K.L., et al. (2000). The LIM homeobox gene Lhx9 is essential for mouse gonad formation. Nature *403*, 909–913. https://doi.org/10.1038/35002622.

Bowles, J., Knight, D., Smith, C., Wilhelm, D., Richman, J., Mamiya, S., Yashiro, K., Chawengsaksophak, K., Wilson, M.J., Rossant, J., et al. (2006). Retinoid signaling determines germ cell fate in mice. Science *312*, 596–600. https://doi.org/10.1126/science.1125691.

Brennan, J., and Capel, B. (2004). One tissue, two fates: molecular genetic events that underlie testis versus ovary development. Nat. Rev. Genet. *5*, 509–521. https://doi.org/10.1038/nrg1381.

Chassot, A.A., Ranc, F., Gregoire, E.P., Roepers-Gajadien, H.L., Taketo, M.M., Camerino, G., de Rooij, D.G., Schedl, A., and Chaboissier, M.C. (2008). Activation of beta-catenin signaling by Rspo1 controls differentiation of the mammalian ovary. Hum. Mol. Genet. *17*, 1264–1277. https://doi.org/10.1093/hmg/ddn016.

Chen, Y.Y., Russo, D.D., Drake, R.S., Duncan, F.E., Shalek, A.K., Goods, B.A., and Woodruff, T.K. (2022). Single-cell transcriptomics of staged oocytes and somatic cells reveal novel regulators of follicle activation. Reproduction *164*, 55–70. https://doi.org/10.1530/rep-22-0053.

Cortvrindt, R., Smitz, J., and Van Steirteghem, A.C. (1996). In-vitro maturation, fertilization and embryo development of immature oocytes from early preantral follicles from prepuberal mice in a simplified culture system. Hum. Reprod. *11*, 2656–2666. https://doi.org/10.1093/oxfordjournals.humrep.a019188.

Dobin, A., Davis, C.A., Schlesinger, F., Drenkow, J., Zaleski, C., Jha, S., Batut, P., Chaisson, M., and Gingeras, T.R. (2013). STAR: ultrafast universal RNA-seq aligner. Bioinformatics *29*, 15–21. https://doi.org/10.1093/bioinformatics/bts635.

Dong, J., Albertini, D.F., Nishimori, K., Kumar, T.R., Lu, N., and Matzuk, M.M. (1996). Growth differentiation factor-9 is required during early ovarian folliculogenesis. Nature *383*, 531–535. https://doi.org/10.1038/383531a0.

Ginsburg, M., Snow, M.H., and McLaren, A. (1990). Primordial germ cells in the mouse embryo during gastrulation. Development *110*, 521–528. https://doi.org/10.1242/dev.110.2.521.

Hacker, A., Capel, B., Goodfellow, P., and Lovell-Badge, R. (1995). Expression of Sry, the mouse sex determining gene. Development *121*, 1603–1614. https://doi.org/10.1242/dev.121.6.1603.

Hayashi, K., Hikabe, O., Obata, Y., and Hirao, Y. (2017). Reconstitution of mouse oogenesis in a dish from pluripotent stem cells. Nat. Protoc. *12*, 1733–1744. https://doi.org/10.1038/nprot.2017.070.

Hayashi, K., Ogushi, S., Kurimoto, K., Shimamoto, S., Ohta, H., and Saitou, M. (2012). Offspring from oocytes derived from *in vitro* primordial germ cell-like cells in mice. Science *338*, 971–975. https://doi.org/10.1126/science.1226889.

Hayashi, K., Ohta, H., Kurimoto, K., Aramaki, S., and Saitou, M. (2011). Reconstitution of the mouse germ cell specification pathway in culture by pluripotent stem cells. Cell *146*, 519–532. https://doi.org/10.1016/j.cell.2011.06.052.

Hayashi, K., and Saitou, M. (2013). Generation of eggs from mouse embryonic stem cells and induced pluripotent stem cells. Nat. Protoc. *8*, 1513–1524. https://doi.org/10.1038/nprot.2013.090.

Hikabe, O., Hamazaki, N., Nagamatsu, G., Obata, Y., Hirao, Y., Hamada, N., Shimamoto, S., Imamura, T., Nakashima, K., Saitou, M., and Hayashi, K. (2016). Reconstitution *in vitro* of the entire cycle of the mouse female germ line. Nature *539*, 299–303. https://doi.org/10.1038/nature20104.

Hirshfield, A.N. (1992). Heterogeneity of cell populations that contribute to the formation of primordial follicles in rats. Biol. Reprod. *47*, 466–472. https://doi.org/10.1095/biolreprod47.3.466.

Hu, Y.C., Okumura, L.M., and Page, D.C. (2013). Gata4 is required for formation of the genital ridge in mice. PLoS Genet. *9*, e1003629. https://doi.org/10.1371/journal.pgen.1003629.

Hunter, N. (2017). Oocyte Quality Control: Causes, Mechanisms, and Consequences. Cold Spring Harb. Symp. Quant. Biol. *82*, 235–247. https://doi.org/10.1101/sqb.2017.82.035394.

Inman, K.E., and Downs, K.M. (2007). The murine allantois: emerging paradigms in development of the mammalian umbilical cord and its relation to the fetus. Genesis *45*, 237–258. https://doi.org/10.1002/dvg.20281.

John, G.B., Gallardo, T.D., Shirley, L.J., and Castrillon, D.H. (2008). Foxo3 is a PI3K-dependent molecular switch controlling the initiation of oocyte growth. Dev. Biol. *321*, 197–204. https://doi.org/10.1016/j.ydbio.2008.06.017.

Kusaka, M., Katoh-Fukui, Y., Ogawa, H., Miyabayashi, K., Baba, T., Shima, Y., Sugiyama, N., Sugimoto, Y., Okuno, Y., Kodama, R., et al. (2010). Abnormal epithelial cell polarity and ectopic epidermal growth factor receptor (EGFR) expression induced in Emx2 KO embryonic gonads. Endocrinology *151*, 5893–5904. https://doi.org/10.1210/en.2010-0915.

Laronda, M.M., Rutz, A.L., Xiao, S., Whelan, K.A., Duncan, F.E., Roth, E.W., Woodruff, T.K., and Shah, R.N. (2017). A bioprosthetic ovary created using 3D printed microporous scaffolds restores ovarian function in sterilized mice. Nat. Commun. *8*, 15261. https://doi.org/10.1038/ncomms15261.

Liao, Y., Smyth, G.K., and Shi, W. (2013). The Subread aligner: fast, accurate and scalable read mapping by seed-and-vote. Nucleic Acids Res. *41*, e108. https://doi.org/10.1093/nar/gkt214.

Liu, C., Peng, J., Matzuk, M.M., and Yao, H.H.C. (2015). Lineage specification of ovarian theca cells requires multicellular interactions via oocyte and granulosa cells. Nat. Commun. *6*, 6934. https://doi.org/10.1038/ncomms7934.

Liu, C.F., Bingham, N., Parker, K., and Yao, H.H.C. (2009). Sex-specific roles of beta-catenin in mouse gonadal development. Hum. Mol. Genet. *18*, 405–417. https://doi.org/10.1093/hmg/ddn362.

Luo, X., Ikeda, Y., and Parker, K.L. (1995). The cell-specific nuclear receptor steroidogenic factor 1 plays multiple roles in reproductive function. Philos. Trans. R. Soc. Lond. B Biol. Sci. *350*, 279–283. https://doi.org/10.1098/rstb.1995.0162.

Merchant, H. (1975). Rat gonadal and ovarioan organogenesis with and without germ cells. An ultrastructural study. Dev. Biol. *44*, 1–21. https://doi.org/10.1016/0012-1606(75)90372-3.

Mork, L., Maatouk, D.M., McMahon, J.A., Guo, J.J., Zhang, P., McMahon, A.P., and Capel, B. (2012). Temporal differences in granulosa cell specification in the ovary reflect distinct follicle fates in mice. Biol. Reprod. *86*, 37. https://doi.org/10.1095/biolreprod.111.095208.

Newman Hirshfield, A. (1991). Development of Follicles in the Mammalian Ovary. Int. Rev. Cytol. *124*, 43–101.

Ng, A., Tan, S., Singh, G., Rizk, P., Swathi, Y., Tan, T.Z., Huang, R.Y.J., Leushacke, M., and Barker, N. (2014). Lgr5 marks stem/progenitor cells in ovary and tubal epithelia. Nat. Cell Biol. *16*, 745–757. https://doi.org/10.1038/ncb3000.

Nicholls, P.K., Schorle, H., Naqvi, S., Hu, Y.C., Fan, Y., Carmell, M.A., Dobrinski, I., Watson, A.L., Carlson, D.F., Fahrenkrug, S.C., and Page, D.C. (2019). Mammalian germ cells are determined after PGC colonization of the nascent gonad. Proc. Natl. Acad. Sci. USA *116*, 25677–25687. https://doi.org/10.1073/pnas.1910733116.

Niu, W., and Spradling, A.C. (2020). Two distinct pathways of pregranulosa cell differentiation support follicle formation in the mouse ovary. Proc. Natl. Acad. Sci. USA *117*, 20015–20026. https://doi.org/10.1073/pnas.2005570117.

Ohinata, Y., Sano, M., Shigeta, M., Yamanaka, K., and Saitou, M. (2008). A comprehensive, non-invasive visualization of primordial germ cell development in mice by the Prdm1-mVenus and Dppa3-ECFP double transgenic reporter. Reproduction *136*, 503–514. https://doi.org/10.1530/REP-08-0053.

Pangas, S.A., Saudye, H., Shea, L.D., and Woodruff, T.K. (2003). Novel approach for the three-dimensional culture of granulosa cell-oocyte complexes. Tissue Eng. *9*, 1013–1021. https://doi.org/10.1089/107632703322495655.

Pepling, M.E., and Spradling, A.C. (1998). Female mouse germ cells form synchronously dividing cysts. Development *125*, 3323–3328. https://doi.org/10.1242/dev.125.17.3323.

Pepling, M.E., and Spradling, A.C. (2001). Mouse ovarian germ cell cysts undergo programmed breakdown to form primordial follicles. Dev. Biol. *234*, 339–351. https://doi.org/10.1006/dbio.2001.0269.

Rastetter, R.H., Bernard, P., Palmer, J.S., Chassot, A.A., Chen, H., Western, P.S., Ramsay, R.G., Chaboissier, M.C., and Wilhelm, D. (2014). Marker genes identify three somatic cell types in the fetal mouse ovary. Dev. Biol. *394*, 242–252. https://doi.org/10.1016/j.ydbio.2014.08.013.

Saitou, M., Barton, S.C., and Surani, M.A. (2002). A molecular programme for the specification of germ cell fate in mice. Nature *418*, 293–300. https://doi.org/10.1038/nature00927.

Schmidt, D., Ovitt, C.E., Anlag, K., Fehsenfeld, S., Gredsted, L., Treier, A.C., and Treier, M. (2004). The murine winged-helix transcription factor Foxl2 is required for granulosa cell differentiation and ovary maintenance. Development *131*, 933–942. https://doi.org/10.1242/dev.00969.

Shimamoto, S., Nishimura, Y., Nagamatsu, G., Hamada, N., Kita, H., Hikabe, O., Hamazaki, N., and Hayashi, K. (2019). Hypoxia induces the dormant state in oocytes through expression of Foxo3. Proc. Natl. Acad. Sci. USA *116*, 12321–12326. https://doi.org/10.1073/pnas.1817223116.

Smith, T., Heger, A., and Sudbery, I. (2017). UMI-tools: modeling sequencing errors in Unique Molecular Identifiers to improve quantification accuracy. Genome Res. *27*, 491–499. https://doi.org/10.1101/gr.209601.116.

Stévant, I., Kühne, F., Greenfield, A., Chaboissier, M.C., Dermitzakis, E.T., and Nef, S. (2019). Dissecting Cell Lineage Specification and Sex Fate Determination in Gonadal Somatic Cells Using Single-Cell Transcriptomics. Cell Rep. *26*, 3272–3283.e3. https://doi.org/10.1016/j.celrep.2019.02.069.

Stuart, T., Butler, A., Hoffman, P., Hafemeister, C., Papalexi, E., Mauck, W.M., 3rd, Hao, Y., Stoeckius, M., Smibert, P., and Satija, R. (2019). Comprehensive Integration of Single-Cell Data. Cell *177*, 1888–1902.e21. https://doi.org/10.1016/j.cell.2019.05.031.

Wilhelm, D., Washburn, L.L., Truong, V., Fellous, M., Eicher, E.M., and Koopman, P. (2009). Antagonism of the testis- and ovary-determining pathways during ovotestis development in mice. Mech. Dev. *126*, 324–336. https://doi.org/10.1016/j.mod.2009.02.006.

Xiao, S., Coppeta, J.R., Rogers, H.B., Isenberg, B.C., Zhu, J., Olalekan, S.A., McKinnon, K.E., Dokic, D., Rashedi, A.S., Haisenleder, D.J., et al. (2017). A microfluidic culture model of the human reproductive tract and 28-day menstrual cycle. Nat. Commun. *8*, 14584. https://doi.org/10.1038/ncomms14584.

Yoshino, T., Suzuki, T., Nagamatsu, G., Yabukami, H., Ikegaya, M., Kishima, M., Kita, H., Imamura, T., Nakashima, K., Nishinakamura, R., et al. (2021). Generation of ovarian follicles from mouse pluripotent stem cells. Science *373*, eabe0237. https://doi.org/10.1126/science.abe0237.

Stem Cell Reports, Volume *18*

Supplemental Information

Reconstituted ovaries self-assemble without an ovarian surface epithelium

Enrique Sosa, Sinthia Kabir Mumu, Christian C. Alvarado, Qiu Ya Wu, Isaias Roberson, Alejandro Espinoza, Fei-man Hsu, Kaori Saito, Timothy J. Hunt, Jared E. Faith, Matthew G. Lowe, Jonathan A. DiRusso, and Amander T. Clark

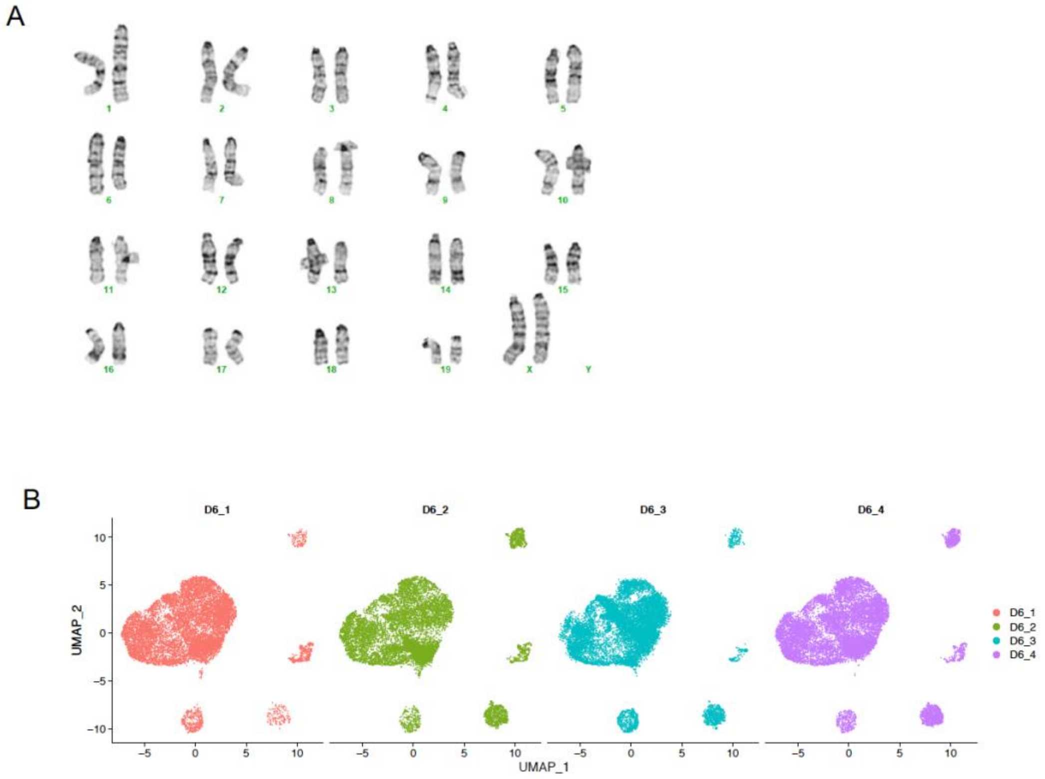

Figure S1: Characterization of PGCLCs and somatic cells. Related to Figure 1.

A) Karyotype with G-banding of the BVSCs iPSC clone 4 FR C3 used for PGCLC and somatic cell differentiation.

B) UMAP of the results from each Day 6 differentiation experiment shown separately indicating equal contribution of cells from each experiment to the five distinct cell populations.

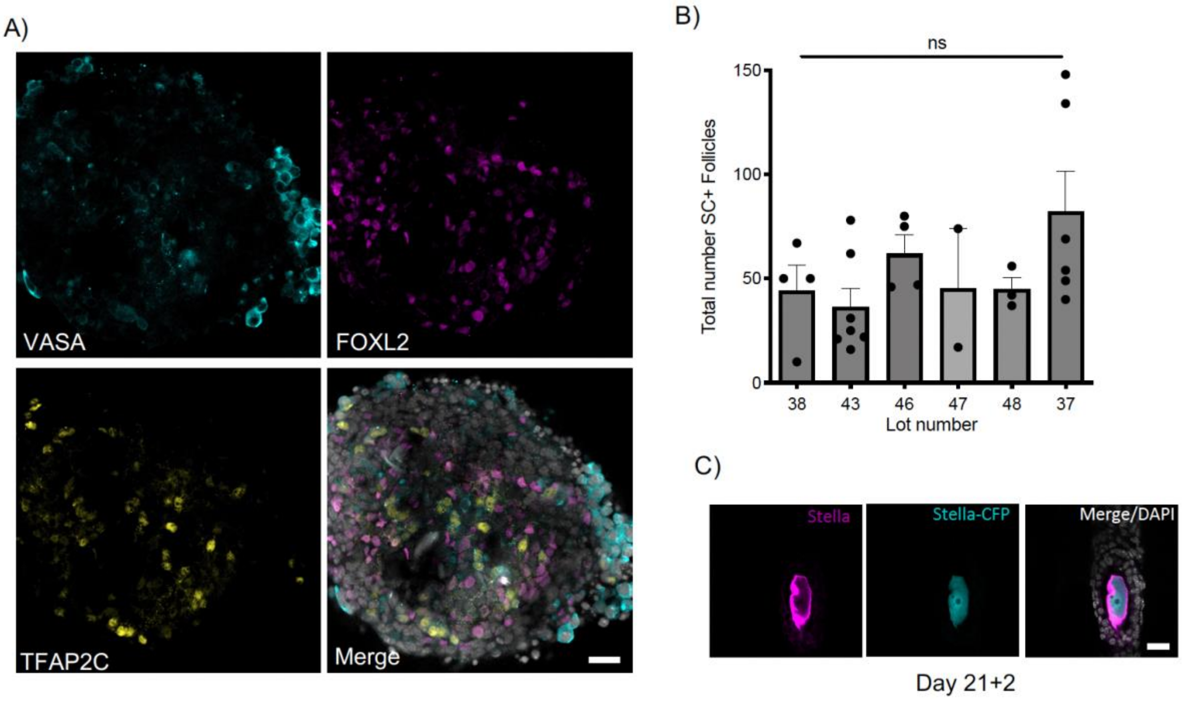

Figure S2: Characterization of rOvaries. Related to Figure 4.

A) IF of aggregates containing FOSCs and PGCLCs at day 2 of aggregation. TFAP2C (yellow) marks the PGCLCs. VASA (blue) marks the endogenous PGCs that escape MACS. FOXL2 (pink) marks pre-granulosa/granulosa cells. Scale bar is 10um.

B) Average number of CFP+ oocytes in rOvaries at the conclusion of IVDi. X-axis refers to different Lots of FOSCs. Each dot refers to the number of SC+ oocytes in a given ovary. Data are shown as mean and standard error of the mean. Statistical analysis in E and F involved an *ordinary one-way ANOVA followed by Tukey's multiple comparison test. Significance was accepted if p<0.05.*

C) IF for STELLA was performed on frozen sections of day 21 rOvaries so that CFP could still be detected. Scale bar is 20um.

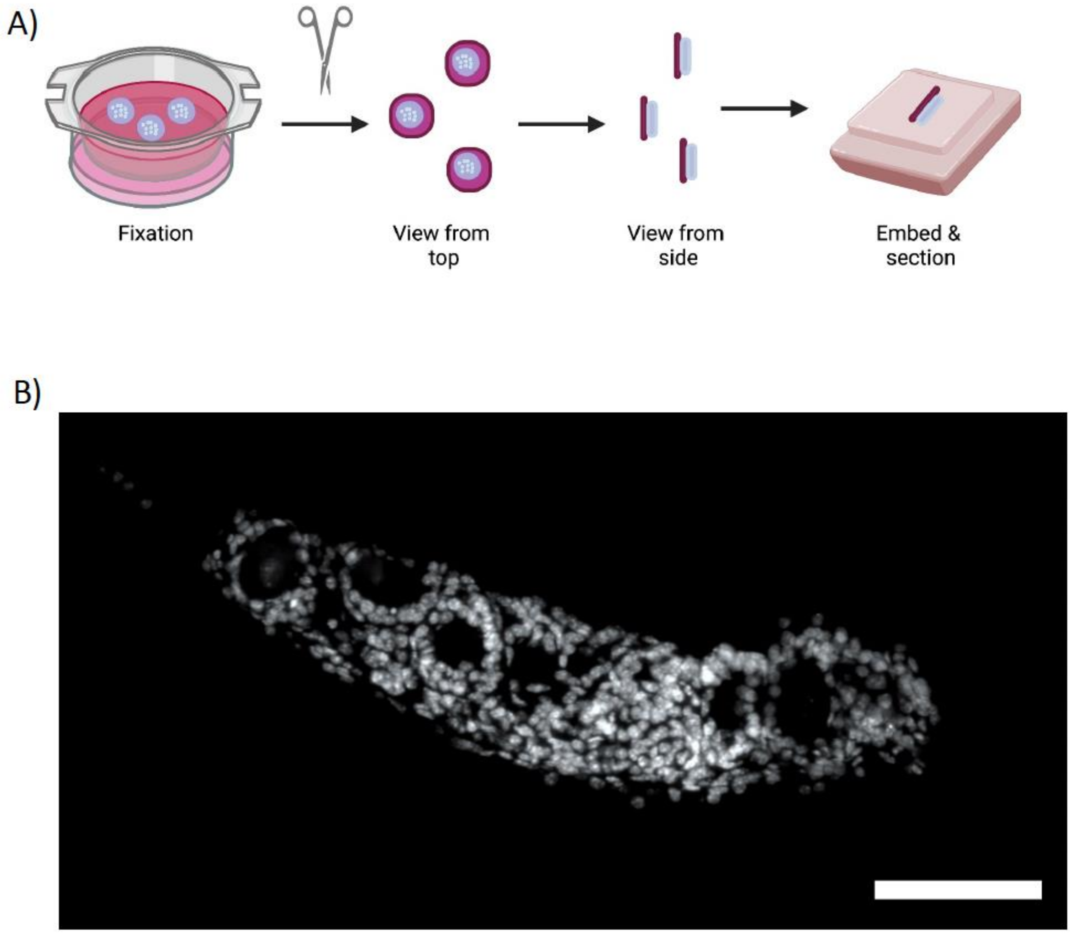

Figure S3: Processing of rOvaries at day 21 of IVDi. Related to Figure 5.

A) Schematic for processing rOvaries at day 21 of IVDi. Created with Biorender.com.
B) DAPI stain of an rOvary showing a view from the side. Scale bar is 100um.

SUPPLEMENTAL TABLES

Table S1: Filtering strategy used to identify valid cells at day 6 of differentiation for further analysis. Related to Figure 1.

Sample	# Reads	Valid Barcodes (%)	# Valid Cells	# Genes per cell	# UMI per cell
D6_1	278,803,599	97.6%	10,419	3,700	10,002
D6_2	1,633,860,575	97.2%	10,290	6,724	41,055
D6_3	179,195,300	97.1	12,289	2,685	6,208
D6_4	109,172,922	97.2	12,194	2,145	4,270

Table S2: Filtering strategy used to identify valid FOSCs for 10X genomics analysis. Related to Figure 2.

FOSC Lot	# Reads	Mappability (%)	# Valid Cells	# Genes per cell	# UMI per cell
Lot 26	389,976,563	76.4	9,668	3,626	11,581
Lot 29	229,173,801	77.4	8,548	3,102	9,322
Lot 30	417,755,755	77.8	10,692	3,686	12,053

Table S3: Percentage and number of Cell types identified in each FOSC Lot. Related to Figure 2.

	Lot 26 (%)	Lot 26 (number)	Lot 29 (%)	Lot 29 (number)	Lot 30 (%)	Lot 30 (number)
Pre-granulosa	46.74%	4519	41.51%	3548	48.77%	5215
Epithelial	17.09%	1652	23.98%	2050	22.40%	2395
Stromal fibroblast	25.19%	2435	25.61%	2189	23.92%	2558
Germ	7.40%	715	3.90%	333	3.41%	365
Endothelial	1.77%	171	1.01%	86	0.80%	86
Macrophage	0.72%	70	0.27%	23	0.41%	44
RBC	0.18%	17	0.40%	34	0.12%	13
Sertoli Cells	0.92%	89	3.33%	285	0.15%	16

Table S4: Total number of CFP-negative oocytes at D21+2 from rOvaries generated with different FOSC Lots. Related to Figure 4.

rOvary	Lot 26	Lot 29	Lot 30	Lot 37	Lot 38	Lot 43	Lot 46	Lot 47	Lot 48
1	9	5	3	11	26	17	11	4	10
2	10	12	9	18	19	6	7	1	9
3	4	13	0	9	26	8	9	1	12
4	10		8	18		5	19	2	
5	8		10	14			14	2	
6	1		13				16	6	
7	5						7		
8	8						10		
9	24						5		
10	12						1		
11	16								

Table S5. List of antibodies used in this study.

Target antigens	Antibodies	Dilutions	Application	Suppliers	Catalog#	RRID
FOXL2	Goat polyclonal anti-FOXL2	1:100	IF	Novus Biologicals	NB100-1277	AB_2106188
KRT19	Rabbit polyclonal anti-KRT19	1:200	IF	Proteintech	10712-1-AP	AB_2133325
LAMININ	Rabbit polyclonal anti-LAMININ	1:200	IF	Abcam	ab11575	AB_298179
NR2F2 (COUP-TF II)	Mouse monoclonal anti-NR2F2	1:200	IF	R&D Systems	PP-H7147-00	AB_2155627
3βHSD	Mouse monoclonal anti-3βHSD	1:200	IF	Cosmo bio	KO607	AB_2722746
TFAP2C (AP-2γ)	Mouse monoclonal anti-AP-2γ	1:200	IF	Santa Cruz Biotech	sc-12762	AB_667770
VASA (DDX4/MVH)	Rabbit poly clonal anti-DDX4/MVH	1:100	IF	Abcam	ab13840	AB_443012

VASA (DDX4)	Goat polyclonal anti-hVASA	1:100	IF	R&D	AF2030	AB_2277369
CFP	Chicken polyclonal anti-GFP	1:200	IF	Abcam	ab13970	AB_300798
Chicken IgY	FITC-conjugated Donkey anti chicken IgY	1:400	IF	Abcam	ab63507	AB_1139472
Mouse IgG	AF488-conjugated donkey-anti-mouse	1:200; 1:400	IF	Jackson ImmunoResearch	715-546-150	AB_2340849
Mouse IgG	AF647-conjugated donkey-anti-mouse	1:200	IF	Jackson ImmunoResearch	715-605-151	AB_2340863
Goat IgG	AF594-conjugated donkey-anti-goat	1:200	IF	Jackson ImmunoResearch	705-586-147	AB_2340434
Rabbit IgG	AF488-conjugated donkey-anti-rabbit	1:200; 1:400	IF	Jackson ImmunoResearch	711-545-152	AB_2313584
SSEA-1(CD15) human, mouse	Anti-SSEA-1(CD15) MicroBeads	1:8	MACS	Miltenyibiotec	130-094-530	AB_2814656
CD31 mouse	Anti-CD31 MicroBeads	1:8	MACS	Miltenyibiotec	130-097-418	AB_2814657
SSEA-1 human, mouse	PE-conjugated Anti-SSEA-1	1:50	FACS	Miltenyibiotec	130-117-689	AB_2728020
